# Know your enemy – transcriptome of myxozoan *Tetracapsuloides bryosalmonae* reveals potential drug targets against proliferative kidney disease in salmonids

**DOI:** 10.1017/S003118202100010X

**Published:** 2021-05

**Authors:** Freed Ahmad, Paul V. Debes, Lilian Pukk, Siim Kahar, Hanna Hartikainen, Riho Gross, Anti Vasemägi

**Affiliations:** 1 Department of Biology, University of Turku, FI-20014, Finland; 2 Department of Aquaculture and Fish Biology, Hólar University, Saudárkrókur, Iceland; 3 Chair of Aquaculture, Institute of Veterinary Medicine and Animal Sciences, Estonian University of Life Sciences, 51014 Tartu, Estonia; 4 School of Life Sciences, The University of Nottingham, University Park, NG7 5RD, Nottingham, UK; 5 Department of Aquatic Resources, Swedish University of Agricultural Sciences, 17893 Drottningholm, Sweden

**Keywords:** Aquatic pathogens, climate change, dual RNA-seq, minicollagen, Myxozoa, next-generation sequencing, salmonid fish

## Abstract

The myxozoan *Tetracapsuloides bryosalmonae* is a widely spread endoparasite that causes proliferative kidney disease (PKD) in salmonid fish. We developed an *in silico* pipeline to separate transcripts of *T. bryosalmonae* from the kidney tissue of its natural vertebrate host, brown trout (*Salmo trutta*). After stringent filtering, we constructed a partial transcriptome assembly *T. bryosalmonae*, comprising 3427 transcripts. Based on homology-restricted searches of the assembled parasite transcriptome and Atlantic salmon (*Salmo salar*) proteome, we identified four protein targets (Endoglycoceramidase, Legumain-like protease, Carbonic anhydrase 2, Pancreatic lipase-related protein 2) for the development of anti-parasitic drugs against *T. bryosalmonae*. Earlier work of these proteins on parasitic protists and helminths suggests that the identified anti-parasitic drug targets represent promising chemotherapeutic candidates also against *T. bryosalmonae*, and strengthen the view that the known inhibitors can be effective in evolutionarily distant organisms. In addition, we identified differentially expressed *T. bryosalmonae* genes between moderately and severely infected fish, indicating an increased abundance of *T. bryosalmonae* sporogonic stages in fish with low parasite load. In conclusion, this study paves the way for future genomic research in *T. bryosalmonae* and represents an important step towards the development of effective drugs against PKD.

## Graphical abstract

**Figure:**
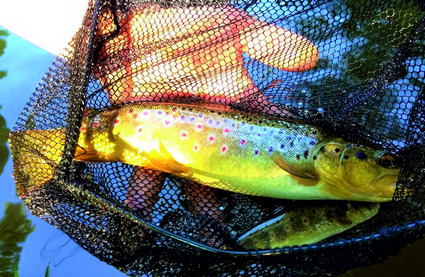

## Introduction

*Tetracapsuloides bryosalmonae* is an obligate endoparasite that causes proliferative kidney disease (PKD) in salmonid fish. PKD is considered as one of the most serious emerging temperature-dependent parasitic freshwater diseases of salmonids in the Northern Hemisphere, causing significant losses on farms and in natural populations (Okamura *et al*., 2001, 2011). *Tetracapsuloides bryosalmonae* belongs to the Class Myxozoa within the Phylum Cnidaria and belongs to the relative species poor Subclass Malacosporea (Giribet and Edgecombe, 2020). The parasite has a two-host life-cycle, alternating between an invertebrate host (several species of freshwater bryozoans) and a fish host (salmonids) (Okamura *et al*., 2011). Fish host invasion occurs *via* epidermal mucous cells (e.g. gills, skin; Longshaw *et al*., 2002), followed by dissemination to internal organs, primarily the kidney and spleen *via* the vascular system. Sporogonic development takes place in the kidney of permissive fish hosts, and spores infective to bryozoans are released in fish urine (Hedrick *et al*., 2004). The development of PKD symptoms is temperature-dependent. Exposure to water temperatures ≥15°C elicits a strong immune response, kidney hypertrophy and anaemia, which may lead to death (Hedrick *et al*., 1993; Bettge *et al*., 2009). As a consequence, the decline of native salmonid fish populations in several countries has been linked with a combination of increasing temperatures and emergence of PKD (Wahli *et al*., 2002; Okamura *et al*., 2011; Sudhagar *et al*., 2020).

Despite the high economic and ecological significance of PKD, the genomic resources for *T. bryosalmonae* are limited to a few hundred expressed sequence tags and some other targeted loci (e.g. Saulnier *et al*., 1996; Holland *et al*., 2011; Hartikainen *et al*., 2014, 2015; Carraro *et al*., 2018). Only very recently, an intersect transcriptome of *T. bryosalmonae* consisting of *de novo* assembled transcripts present in both rainbow trout (*Oncorhynchus mykiss*) and bryozoan host (*Fredericella sultana*) has been reported (Faber *et al*., 2021). However, the *T. bryosalmonae* transcriptome recovered from the dead-end host *O. mykiss* might lack genes essential for the parasite to reach sporogony in natural fish host. Knowledge of the parasite genomic composition and gene expression is therefore needed to better understand the underlying mechanisms of parasite life stages, host–parasite interactions, resistance, tolerance and virulence. Moreover, genomic information may help to predict potential new drug targets and drugs/drug-like compounds against the parasite (Cowell and Winzeler, 2019). The climate-change driven emergence of PKD underlies the urgency for the identification of effective chemotherapeutic targets against *T. bryosalmonae* that can be used to protect stocks and improve the welfare of farmed salmonids.

In this study, we report the *de novo* assembly and annotation of the partial *T. bryosalmonae* transcriptome based on dual RNA sequencing (Westermann *et al*., 2012) of both parasite and host utilizing infected brown (*Salmo trutta*) trout kidney tissue. We also identify *T. bryosalmonae* genes that are differentially expressed in relation to parasite load (PL) to obtain a better understanding of host–parasite interactions. Based on searches against known drug target proteins in the ChEMBL database, we identify four promising protein drug targets (consisting of six genes) whose suppression might obstruct the parasite growth and mitigate infections. Thus, these findings prime future experimental approaches to develop and test new control strategies against PKD. Furthermore, the assembled *T. bryosalmonae* transcriptome will be useful in understanding the parasite biology and serve as a valuable reference for characterizing the parasite development during infection.

## Materials and methods

### Sampling and measurement of kidney hyperplasia

Sampling and phenotyping methods of juvenile (0+) brown trout used in this study were reported in Debes *et al*. (2017). DNA was extracted from kidney tissue as in Aljanabi and Martinez (1997). Fourteen fish kidneys corresponding to eight infected and six uninfected fish from three wild populations were selected. All the infected fish originated from River Mustoja (Estonia) and were collected in September, 2014, whereas the uninfected fish originated from two parasite-free rivers (Preedi and Vodja, Estonia; three per river) and were collected in September, 2015. Sampling from multiple populations was necessary as *T. bryosalmonae* prevalence in the study area is often 100% (Dash and Vasemägi, 2014; Debes *et al*., 2017), prohibiting the sampling of both infected and uninfected individuals from the same location. We also quantified a typical disease trait associated with PKD, kidney swelling (renal hyperplasia), estimated as a body-length-standardised kidney depth, as described by Debes *et al*. (2017).

### PL measurement

PL (a proxy for the inverse of resistance) was determined from infected fish kidney tissue by qPCR using previously described protocols (Bruneaux *et al.* 2016; Debes *et al.* 2017). For each sample, we quantified the occurrence of two target DNA sequences: a 166 bp fragment of *T. bryosalmonae* 18S rDNA sequence (GenBank Accession U70623) and 74 bp fragment of the *S. salar* nuclear DNA sequence (GenBank Accession BT049744.1, Prefoldin subunit 6). Simultaneous quantification of both DNA fragments enabled us to standardize the amount of parasite DNA relative to host DNA. Three technical replicates were run for both targets in each sample. Based on PL, the infected samples were divided into categories of moderate (*n* = 4) and severe (*n* = 4) infection. On average the moderately infected fish exhibited lower kidney swelling measurements, compared to severely infected fish (~twice the kidney depth; Supplementary Fig. 1).

### RNA isolation, library preparation and RNA sequencing

We preserved half of the kidney cross-section for RNA isolation by freezing them in liquid N_2_ and storing them at −80°C. The other half of the kidney cross-section was stored in the same way but used for estimation of PL using qPCR. Total RNA was extracted from the kidney cross-section of the 14 individuals using NucleoSpin^®^ RNA kit. RNA quality was tested using the Advanced Analytical Fragment Analyzer. Quantity of each total RNA sample was measured with a Nanodrop ND-2000 spectrophotometer. Library preparation was done according to Illumina TruSeq^®^ Stranded mRNA Sample Preparation Guide (part # 15031047) starting from 100 ng of total RNA. The steps involved in cDNA library constructions were as follows: purification of poly-A containing mRNA, fragmentation of mRNA using divalent cations, first-strand (using reverse transcriptase and random primers) and second strand (using DNA Polymerase I and RNase H) synthesis of cDNA, purification and enrichment with PCR. A unique Illumina TruSeq indexing adapter was ligated to each sample during the adapter ligation step to be able to pool several samples for sequencing. The quality of the prepared libraries was evaluated with Advanced Analytical Fragment Analyzer. The concentration of each library was quantified with Qubit^®^ Fluorometric Quantitation, Life Technologies. Due to adapter peaks, the libraries were purified twice. The samples were normalized and pooled for the automated cluster preparation that was carried out using Illumina cBot station. The 14 libraries were pooled into a single pool. The samples were sequenced with Illumina HiSeq 2500 instrument using TruSeq v3 sequencing chemistry in three lanes. Paired-end sequencing with 100 bp read length was used, followed by 6 bp index run. The base calling was performed using Illumina's standard bcl2fastq2 software. RNA sequencing was performed at the Finnish Functional Genomics Centre, Turku Centre for Biotechnology.

### Data processing and removal of host RNA sequence

The quality assessment of sequenced reads was performed with FastQC 0.10.1 (http://www.bioinformatics.babraham.ac.uk/projects/fastqc/) both before and after pre-processing. Trimmomatic (v. 0.35) was used for adapter trimming and read pre-processing (parameters: ILLUMINACLIP = TruSeq3-s.e..fa; HEADCROP = 10; SLIDINGWINDOW = 4:20; LEADING = 5; TRAILING = 5; MINLEN = 40). In order to assemble a host-free parasite transcriptome, the filtered reads were first mapped to the Atlantic salmon genome (GCF_000233375.1) and the unmapped reads were subsequently mapped to the northern pike (*Esox lucius*, Esociformes) genome (GCF_000721915.2) using Hisat2 (v. 2.0.1; score-min = L,0,−0.4) (Kim *et al*., 2015). We used chromosome level assembly of Atlantic salmon genome (Lien *et al*., 2016), as it is evolutionarily close to brown trout (Ahmad *et al*., 2018; Lemopoulos *et al*., 2018), and at the time of analysis, genome assembly of *S. trutta* was not available. Esociformes represents the closest order to Salmoniformes (Rondeau *et al*., 2014). The paired-end reads from the infected fish samples that were not aligned to either the salmon or pike genome were further taxonomically classified by using Kraken2 (Wood *et al*., 2019) with a custom database. The custom Kraken2 database consisted of two fish genomes (Atlantic salmon and Northern pike), four available myxozoan genomes (*Enteromyxum leei*, *Sphaeromyxa zaharoni*, *Kudoa iwatai* and *Thelohanellus kitauei*), both host (*Salmo trutta*, 3177 sequences, mean GC content = 43.2%) and parasite (*T. bryosalmonae*, 741 sequences, mean GC content = 33.6%) nucleotide sequences available in NCBI and ENA (Supplementary Table 1), as well as, unpublished contigs for *T. bryosalmonae,* derived from its bryozoan host *F. sultana* (mean GC content = 27.1%; H. Hartikainen unpublished), along with Kraken2-provided archaea, bacteria, plasmid, viral, human, fungi, protozoa (protists) and UniVec_Core sequences. The reads that were classified within the NCBI bony fish division were excluded before the *de novo* assembly of the parasite transcriptome.

### De novo transcriptome assembly of *T. bryosalmonae*

The putative parasite transcriptome was assembled using Trinity (v. 2.5.1) assembler (Grabherr *et al*., 2011) with the default parameters. CD-HIT-EST (Fu *et al*., 2012) was used to cluster 95% identical transcripts and isoforms. The paired-end reads from both the uninfected samples that were not mapped to any fish genome and the non-bony fish reads from the infected samples were aligned on the clustered transcripts using Bowtie2 (Langmead and Salzberg, 2012). The read count matrix for all samples was generated using the summarize Overlaps function implemented in R for further evaluation and differential expression analyses. In order to filter out transcripts originating from the host or from potential contamination, the longest isoforms of the assembled transcripts were subjected to Kraken2 classification and similarity searching using BLASTN and BLASTX (Altschul *et al*., 1990) against NR databases (only the top hits, dust = no, soft masking = false, *E*-value cut-off = 1 × 10^−4^).

### Filtering and completeness assessment of the parasite transcriptome

The putative parasite transcripts were filtered using the following criteria: (i) at least 40 reads mapped, (ii) 90% or more of the mapped reads originate from infected fish and (iii) retained only those transcripts that matched to GenBank's invertebrate division or no hit was found using Kraken2, BLASTN and BLASTX. In order to assess the completeness of the transcriptome, these transcripts were subjected to Benchmarking Universal Single-Copy Orthologs (BUSCO,v. 3.0) (Simão *et al*., 2015) analysis against 978 Metazoan single-copy orthologs.

Recently, Faber *et al*. (2021) reported an intersect transcriptome of *T. bryosalmonae*, common to both rainbow trout (*O. mykiss*) and bryozoan host (*F. sultana*) which consisted of 7362 contigs showing 50.1% completeness (excluding fragmented hits) against the BUSCO eukaryotic dataset (303 genes). Underestimation of completeness for the myxozoan genomes/transcriptomes using BUSCO metazoan set has already been reported (Hartigan *et al*., 2020; Yahalomi *et al*., 2020). Therefore, in order to identify BUSCO metazoan genes that are either entirely missing or present in all myxozoans, we analysed genome annotations-based coding sequences (CDS) and transcriptome assemblies for seven species: *Thelohanellus kitauei*, *Kudoa iwatai*, *Henneguya salminicola*, *Myxobolus squamalis*, *Myxobolus cerebralis*, *Sphaerospora molnari* and *Ceratonova shasta* (Supplementary Table 2). The *T. kitauei* transcriptome assembly was not available and therefore only CDS extracted from the genome using available genome feature file (GFF) were used. For *K. iwatai,* the genome annotation was not available and therefore, only the transcriptome reads were aligned to the genome using splicing-aware HISAT2 followed by Stringtie (Pertea *et al*., 2015) to assemble alignment into potential transcripts (reference guided transcriptome assembly). The splicing events in the *K. iwatai* genome were summarized using ASTALAVISTA web server (Foissac and Sammeth, 2007). The assembled transcripts were then extracted from the genome using gffread (Pertea and Pertea, 2020). BUSCO genes were considered as present in myxozoans even if it was a fragmented match.

### Annotation of putative parasite transcripts

The functional annotation of the parasite transcripts was performed using two sources, namely; KEGG automatic annotation server (KAAS; Moriya *et al*., 2007) and Trinotate (Bryant *et al*., 2017). For the KEGG Orthology search, the nucleotide sequences of these transcripts were searched against a manually curated set of 39 species (including three cnidarians; *Nematostella vectensis*, *Exaiptasia pallida* and *Hydra vulgaris*). The assigned KEGG ortholog (KO) identifiers were searched for pathways in the reference, as well as, the organism specific pathways (for the three cnidarians) using KEGG mapper (https://www.genome.jp/kegg/tool/map_pathway1.html). The assigned KOs were also tested for pathway enrichment against all the reference KOs using the enrichKEGG (minGSSize = 1, maxGSSize = 5000, other default parameters) function implemented in clusterprofiler. Organism specific pathway enrichment using KO identifiers was not possible in clusterprofiler.

Open reading frames (ORFs; minimum of 50 amino acid length) for the parasite transcripts were identified using Transdecoder (Haas *et al*., 2013) using homology searches (BLASTP and hmmscan (Eddy, 2011) results of longest ORFs against NCBI refseq database and pfam, respectively). The reduced value of minimum ORF length was used to consider the shorter transcripts (200–300 bp, *n* = 526) and fragmented genes for the subsequent annotation. Because myxozoans are underrepresented in the public databases and difficult to annotate, ORFs > 150 bp length (50 aa) were retained even when no homology was found. The resulting protein sequences were then used for BLASTP searches and for signal peptide prediction in Trinotate annotation suite. For Trinotate annotation (report *E*-value <1 × 10^−3^), we used the results from BLASTP and BLASTX homology searches against Swissprot, transmembrane helices predictions using TMHMM Server (v. 2.0), pfam domain search using hmmscan, signal peptides prediction using SignalP 5.0, and BLASTP homology search against Refseq proteins. For the genes with multiple isoforms and protein translations, only the hits with the maximum number of GO terms were reported. Finally, the ribosomal RNAs were predicted using RNAmmer 1.2 Server (Lagesen *et al*., 2007).

### Overlap between two T. bryosalmonae transcriptomes

In order to find the overlap between our *T. bryosalmonae* transcriptome and the recently reported *T. bryosalmonae* intersect transcriptome (from the dead-end vertebrate host, rainbow trout [*O. mykiss*], and the bryozoan *F. sultana;* Faber *et al*., 2021), we carried out reciprocal BLASTN. A transcript (longest isoform in case of multiple isoforms) was considered as present in Faber *et al*. (2021) transcriptome if its top hit (95% minimum identity) matched the best hit in the reciprocal BLASTN. As the scaffolds in Faber *et al*. (2021) transcriptome were relatively long (N50 = 2479) compared to ours (N50 = 657), for the reciprocal BLASTN, we allowed up to 3 hits per scaffold when Faber *et al*. (2021) transcriptome was queried against our transcripts. All isoforms of the same gene were considered as present if the longest isoform was present in Faber *et al*. (2021) transcriptome and vice versa. The GO terms between the genes found in Faber *et al*. (2021) transcriptome *vs* the genes unique to our study were compared using WEGO 2 (Ye *et al*., 2018).

### Differential gene expression analysis of the parasite transcripts

In order to identify potential links between differences in gene expression and parasite development, gene-level differential expression (DE) analysis of *T. bryosalmonae* between the moderately and severely infected fish was carried out using the DEseq function implemented in the R package DESeq2 (Love *et al*., 2014). For this purpose, the read counts of the different isoforms of the same genes were summed up to obtain gene-level read counts. An explorative default significance level (FDR <0.1) was used to identify differentially expressed genes since we were mostly interested in detecting candidates that need further validation using RT-qPCR.

### Identification of drug targets

In order to screen for potential drug targets, *T. bryosalmonae* transcriptome was searched against ChEMBL protein sequences using TBLASTN (soft masking = false, *E*-value cut-off = 1 × 10^−3^). To identify parasite-specific targets and avoid potential drug toxicity to the host, only those transcripts lacking BLASTX hits against the *Salmo salar* proteome (soft masking = false, *E*-value cut-off = 1 × 10^−3^) were retained. To increase the sensitivity of the homology search, we focused on targets with an alignment length >100 amino acids based on TBLASTN search. The protein sequences of the putative parasite transcripts corresponding the top matched drug targets (based on E-value) were subjected to homology-based structure modelling in Phyre2 (http://www.sbg.bio.ic.ac.uk/phyre2) using the intensive mode (Kelley *et al*., 2015). Only those sequences were subject to homology modelling which had a conserved alignment at active or binding sites of the drug targets. The best-supported structures were subsequently superimposed with the template structures using Chimera (v. 1.14; Pettersen *et al*., 2004) for visualization.

## Results

After the quality control and filtering, 493.1 Million (401.6 M paired-end and 91.5 M single-end) reads from the host and parasite were retained (range 22.9–47.2 M reads per sample). Among these, 266 M (220.7 M paired-end and 45.3 M single-end) reads originated from infected fish. After mapping on fish reference genomes, a total of 7.6 M out of 220.7 M (3.4% of the total) paired-end reads from infected fish, that were not assigned to bony fishes by Kraken2, were assembled into 16499 transcripts (12676 genes) using Trinity. Individual transcripts with 95% or more similarity were clustered into 14741 merged transcripts (12621 genes) using CD-HIT-EST with default parameters (Table 1).

**Table 1.** Summary statistics of the *de novo* transcriptome assembly of *T. bryosalmonae*

	All assembled transcripts	Clustered transcripts	Putative parasite transcripts
Total transcripts (genes)	16 499 (12676)	14 741 (12 621)	3427 (2905)
GC content (%)	36.77	36.38	31.50
Contig N50	441	426	657
Median contig length	304	300	483
Average contig length	411.11	402.56	587.07
Total assembled bases	6 782 897	5 934 156	2 011 902
Contig N50 (longest isoform)	414	414	647
Median contig length (longest isoform)	298	298	477
Average contig length (longest isoform)	395.78	396.1	580.75
Total assembled bases (longest isoform)	5 016 954	4 999 154	1 687 084

The overall average alignment rates of infected and uninfected samples against merged transcripts were 83.73 and 33.24%, respectively. Because parasite reads should originate only from infected fish, we used this criterion as an additional step to filter out host transcripts. More specifically, if >10% of the aligned reads of the particular transcript originated from the uninfected fish, the transcript was not assigned as a putative parasite sequence.

**Fig. 1.**
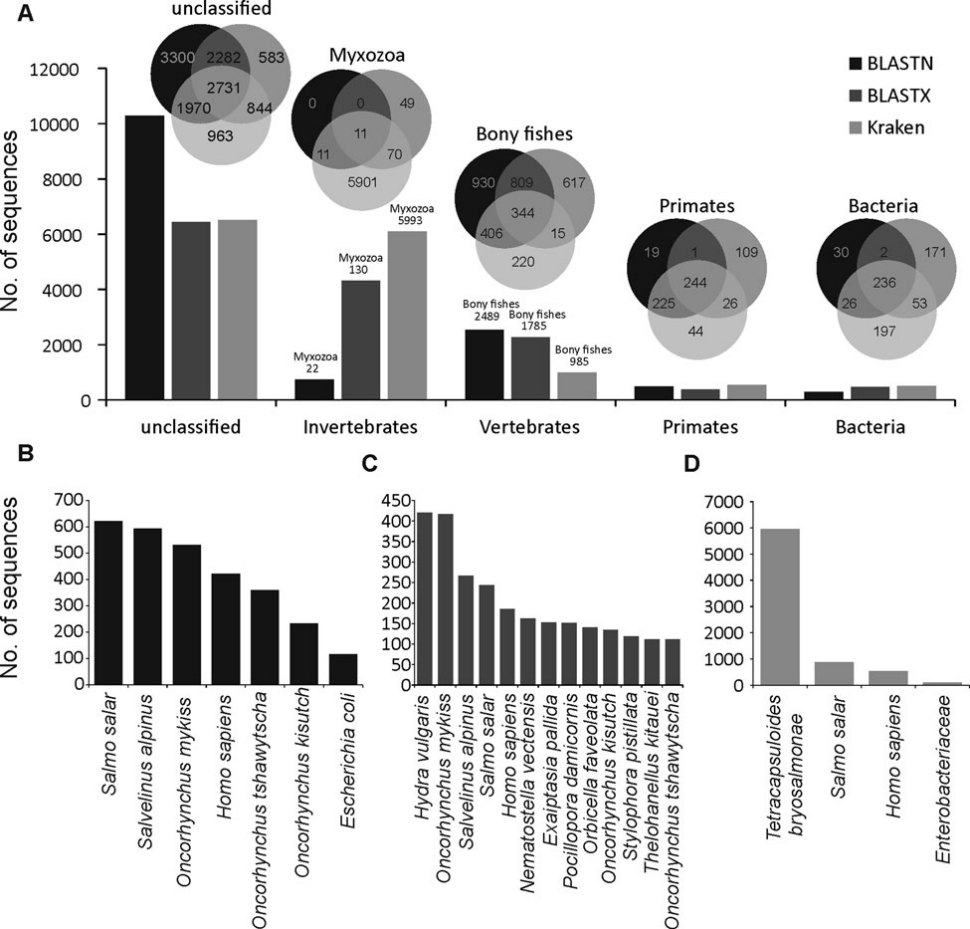
Summary of the taxonomic classification of assembled transcripts. Bar plots in (A) indicate the number of transcripts assigned (or unclassified) to the most frequent GenBank's taxonomic divisions. Venn diagrams illustrate the classification agreement between the BLASTN, BLASTX and Kraken based assignments. Within the invertebrates and vertebrates, only myxozoans and bony fish are shown in the Venn diagrams, respectively. The bar plots in (B–D) show the species to which most of the transcripts were assigned by BLASTN, BLASTX and Kraken, respectively.

A large proportion of merged transcripts were not assigned to any taxonomic group (Fig. 1A, Kraken2 = 6508, BLASTN = 10283 and BLASTX = 6440) reflective of the highly diverged nature of myxozoans from other cnidarians (Chang *et al*., 2015) and a small number of available myxozoan genomes. Among the sequences that were classified using BLASTN, the majority of hits were from vertebrate and salmonid fishes (Fig. 1B). However, relatively more hits to cnidarians (*Hydra vulgaris* and *Nematostella vectensis* and *Exaiptasia pallida*) were identified using BLASTX (Fig. 1C). Only 5993 (40%), 130 (0.9%) and 22 (0.2%) transcripts were assigned to Myxozoa by Kraken2 (Fig. 1D), BLASTX and BLASTN, respectively. Among these, 11 transcripts (Fig. 1A) were designated as myxozoan by all three searches and consisted of following *T. bryosalmonae* genes: antigen PKX101 (seven transcripts including four isoforms of the same gene), minicollagen (Ncol-1, DN1177_c0_g1_i1 and DN14479_c0_g1_i1), DNA repair protein RAD51-like protein (DN15328_c0_g1_i1, excluded because of low coverage) and 60S ribosomal protein L18 (RP-L18, DN3431_c0_g1_i1). BLASTN also assigned some additional transcripts to known genes of *T. bryosalmonae*, including 28S large subunit ribosomal RNA gene (DN11757_c1_g2_i1 and DN11757_c1_g4_i1), S-adenosylmethionine synthetase (DN7631_c0_g1_i1) and ribosomal protein rpl23a-like gene (DN1241_c0_g1_i1), respectively.

### Transcriptome characterization and completeness

**Fig. 2.**
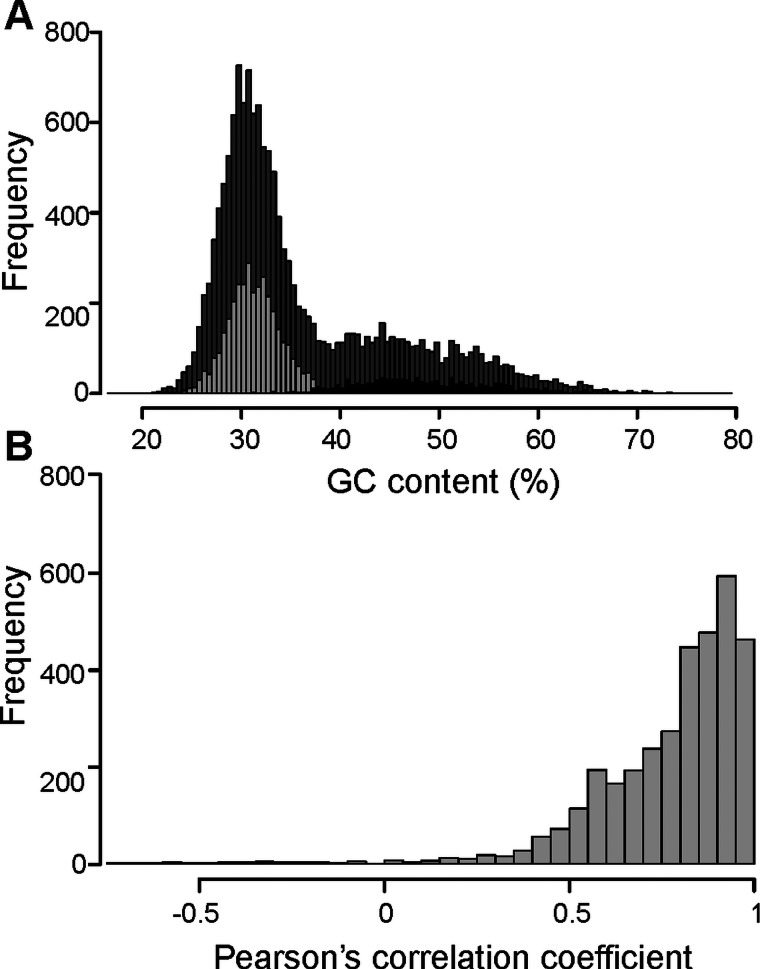
Characterization of *T. bryosalmonae* transcripts: (A) The distributions of GC content of all assembled transcripts (grey; *n* = 14741; mean GC = 35.51%), putative parasitic sequences (light grey; *n* = 3427; mean GC = 31.67%) and transcripts belonging to bony fishes in BLAST results (black; *n* = 1027; mean GC = 48.56%). (B) The distribution of Pearson's correlation coefficients between the raw read counts of putative parasite transcripts and the qPCR-inferred relative parasite load.

The overall distribution of GC content of 14741 merged transcripts was bimodal (Fig. 2A), suggesting that the vertebrate host and myxozoan parasite genomes have different GC content. Previous studies have identified myxozoan transcriptomes and genomes to be predominantly AT-rich (Yang *et al*., 2014; Foox *et al*., 2015; Yahalomi *et al*., 2020). After filtering, the putative parasite transcripts (Fig. 2A; *n* = 3427; mean GC = 31.5%), from here on referred to as *T. bryosalmonae* transcriptome, and transcripts belonging to bony fishes were well separated (Fig. 2A; *n* = 1027; mean GC = 48.56%). The GC content of the *T. bryosalmonae* transcriptome was similar to other myxozoan genomes (GC content 23.6–37.5%) (Yahalomi *et al*., 2020). The GC content of brown trout (43.9%) (Malachowicz *et al*., 2017) also broadly matched with the GC content of the transcripts belonging to bony fishes. The *T. bryosalmonae* transcriptome consisted of more than 2 million bases with an average transcript length of 587 bp (range: 201–3388 bp). Among these transcripts, 929 represented isoform reconstructs of 407 genes, while the remaining 2498 transcripts corresponded to genes without isoforms. The read counts for the large majority of the putative parasite transcripts were positively correlated with the independent estimate of relative PL obtained *via* qPCR assay (Fig. 2B; 92.3% of transcript *r*  ≥ 0.5) suggesting that our bioinformatic pipeline (Supplementary Fig. 2) successfully retained *T. bryosalmonae* reads. Moreover, the positive correlation estimates suggested that the coverage of the majority of assembled transcripts was sufficient to accurately reflect the relative PL.

**Fig. 3.**
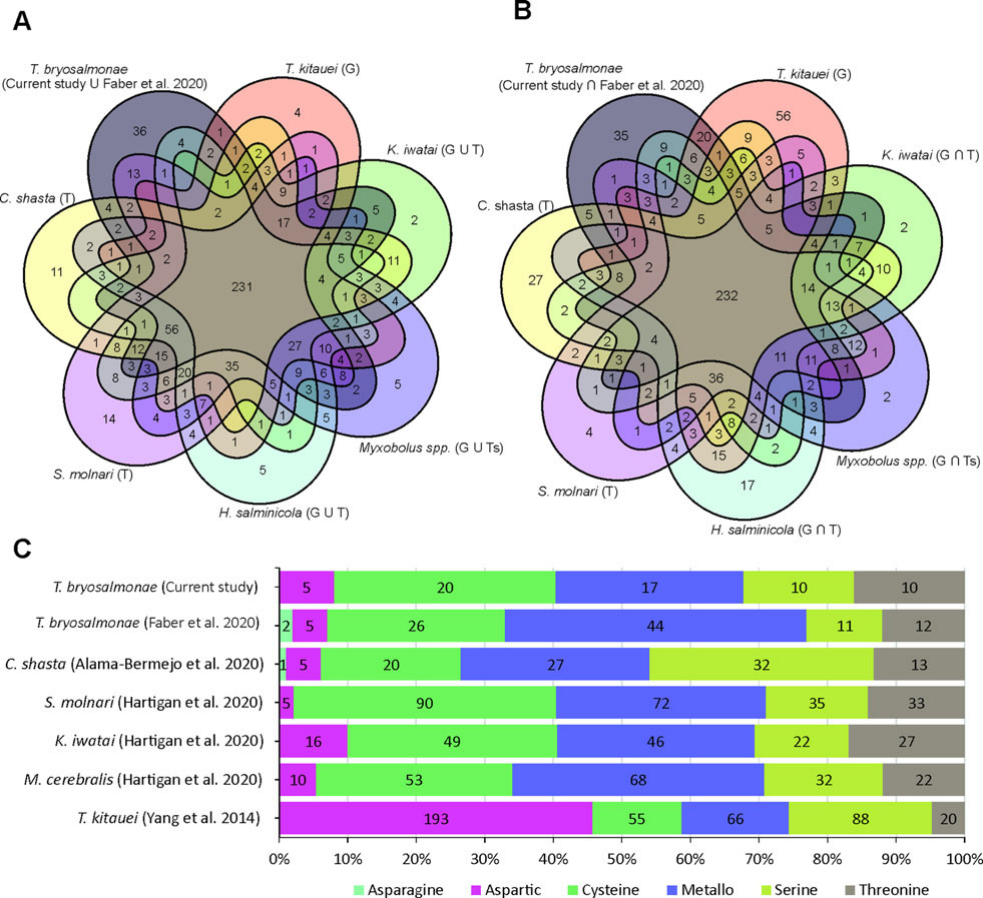
BUSCO single-copy metazoan genes and proteases in Myxozoa (A) Venn diagram showing the number of BUSCO single-copy genes identified (complete + fragmented) in each species. The T and G after the name of species represent *de novo* assembled transcriptome and CDS extracted from the genomes, respectively. (B) Venn diagram showing the number of BUSCO single-copy genes missing in each species. (C) Distribution of proteases in current *T. bryosalmonae* transcriptome in relation to earlier studies. The numbers in each category represent protease classes found in each study.

The assessment of *T. bryosalmonae* transcriptome completeness using BUSCO analysis indicated that only 26% of the core single copy metazoan genes were completely (14.5%) or partially (11.6%) present in our assembly (Supplementary Table 2 and 3). The recently published *T. bryosalmonae* transcriptome (Faber *et al*., 2021) on the other hand exhibited two times higher completeness as 48.3% of the metazoan single copy genes were completely (38.1%) or partially (10.1%) present. A total of 58 BUSCO genes (32 complete single; 2 complete duplicated and 24 fragmented) were identified in our *T. bryosalmonae* transcriptome assembly which were missing in the published transcriptome Faber *et al*. (2021). A similar pattern of relatively low BUSCO completeness can be observed in other published myxozoan transcriptome and genome annotations (Hartigan *et al*., 2020; Yahalomi *et al*., 2020). We subsequently evaluated the presence and absence of BUSCO core single copy metazoan genes in the published myxozoan parasite datasets (Fig. 3A–B, Supplementary Table 3). Despite variation in coverage and completeness of individual transcriptomes and genomes, we identified a set of BUSCO metazoan genes found in all myxozoans (*n* = 231, Fig. 3A). Furthermore, we observed a set of BUSCO metazoan genes (*n* = 232, 23.7%) that were missing in all seven myxozoan transcriptome and genome annotations. This suggests that these genes are either missing or highly divergent in myxozoans (Fig. 3B).

### KEGG ortholog assignment and ribosomal RNA

Out of 3427 putative parasite transcripts, only 1742 transcripts (1509 genes) were assigned to 1172 unique KEGG orthologs (KO) genes, most of which belong to enzymes (657 transcripts; 38%), membrane trafficking (243 transcripts; 14%), spliceosome (206 transcripts; 12%), exosome (173 transcripts; 10%) and chromosome and associated proteins (165 transcripts; 9%) (Supplementary Table 4). Among the enzymes, which were the most abundant proteins, 14% and 11% were protein kinases and peptidases (proteases) and inhibitors, respectively (Supplementary Table 4). The proteases were further distributed into Cysteine (32%), Metallo (27%), Threonine (16%), Serine (16%), and Aspartic proteases (8%) (Supplementary Table 5). Even though the Aspartic proteases were the least numerous proteases (5 genes), their expression was higher than for other protease classes, suggesting potential functional importance. Some of the proteases with the highest expression (average FPKM >1000) were: cathepsin D (CTSD, 2 genes), puromycin-sensitive aminopeptidase (NPEPPS), cytosol aminopeptidase (LAP3) and mitochondrial-processing peptidase subunit beta (PMPCB).

In both reference and cnidarian-specific pathway searches, a large proportion of these transcripts were assigned to metabolic pathways (ko01100; 262 transcripts), spliceosome (ko03040; 141 transcripts), ribosome (ko03010; 74 transcripts), RNA transport (ko03013; 72 transcripts), endocytosis (ko04144; 59 transcripts), oxidative phosphorylation (ko00190; 54 transcripts) and phagosome (ko04145; 54 transcripts) (Supplementary Table 6, Supplementary Fig. 3A). Similar to what has been found for *T. kitauei* (Yang *et al*., 2014), many *T. bryosalmonae* transcripts were assigned to genes involved in multicellular metazoan signalling pathways e.g. mTOR (40 transcripts) and Wnt (22 transcripts) signalling pathways (Supplementary Table 6). The reference-based pathway search also assigned genes to different human disease pathways, which, however, were not observed during organism-specific searches (Supplementary Fig 3B). The enrichment analysis against reference pathways revealed that disease-related genes are enriched among our transcripts and there is an overlap of genes in different disease-related pathways (Supplementary Fig. 4). The excess of human disease-related genes have also been reported in the cnidarian starlet sea anemone (*N. vectensis*) (Sullivan and Finnerty, 2007), which most likely indicates that disease-associated genes in humans are biased towards ancient genes (Domazet-Loso and Tautz, 2008).

Among the transcripts with KO gene assignment, 430 transcripts corresponded to 197 gene isoforms. *Tetracapsuloides bryosalmonae* genes with multiple isoforms included cyclin-dependent kinase 5 (CDK5; 5 isoforms), annexin A7/11 (ANXA7_11; 4 isoforms), ATPase family AAA domain-containing protein 3A/B (ATAD3A_B; 4 isoforms), histone-binding protein RBBP4 (RBBP4; 4 isoforms), SWI/SNF-related matrix-associated actin-dependent regulator of chromatin subfamily A member 2/4 (SMARCA2_4; 4 isoforms), cyclin T (CCNT; 4 isoforms), electron-transferring-flavoprotein dehydrogenase (ETFDH; 4 isoforms), V-type H + -transporting ATPase subunit H (ATPeV1H; 4 isoforms). Among the five CDK5 isoforms, three have premature stop codons while the other two were partial sequences. Similarly, two of the ANXA7_11 isoforms included premature stop codons. The genome-guided transcriptome assembly of *K. iwatai* (assembled for BUSCO, 23896 transcripts, 10556 genes) enabled us to assess the genome-wide pattern of splicing events in the myxozoans. In *K. iwatai,* a total of 23130 splicing events occurred in 5533 genes and intron retention was more frequent (6826, 29.5%) followed by alternative acceptor sites (3437, 14.8%), alternative donor sites (2861, 12.4%) and exon skipping (952, 4.1%). Multiple isoforms of the above-mentioned *T. bryosalmonae* genes (except ETFDH) were also observed in *K. iwatai* reference guided transcriptome assembly (Supplementary Table 7) further confirming that these isoforms are not the artefacts of *de novo* assembly. Thus, the presence of different isoforms may also contribute in increasing the functional diversity of the encoded proteins in Myxozoa. However, further experimental studies are needed to assign and validate the functions of different isoforms of *T. bryosalmonae* genes.

Only a single ribosomal RNA (DN11757_c1_g4_i1) was predicted from our parasite transcripts using RNAmmer showing >2000 bp alignment overlap (99% BLASTN identity) with the partial 28S large subunit ribosomal RNA gene of *T. bryosalmonae* (FJ981822.1).

### Protein coding transcripts and GO annotation

A total of 4046 protein sequences were initially produced from the translation of the parasite transcriptome, which is higher than the number of input transcripts (3324) because multiple protein sequences per transcripts were produced due to short minimum protein length (50 aa). These included; 467 complete (11.5%, mean length 153.8 aa), 12095’- partial (missing start codon) (29.9%; mean length 157.6 aa), 1847 internal (45.6%; mean length 171.6 aa) (missing both start and stop codons) and 5233’- partial (missing stop codon) proteins (12.9%; mean length 143.7 aa).

By employing these sequences in the Trinotate annotation pipeline, a total of 1944 genes were assigned to 25195 GO terms comprising; 11325 (45%) biological processes, 7955 (31.6%) cellular component and 5915 (23.5%) molecular functions. The most abundant GO terms for cellular component, biological process and molecular function included integral component of the membrane, protein phosphorylation and ATP binding, respectively (Supplementary Fig. 5). The complete Trinotate annotation report along-with KEGG ortholog gene identifiers and transcript and protein sequences are provided in Supplementary Table 8.

### Comparison between the two *T. bryosalmonae* transcriptomes

Using an adjusted reciprocal BLASTN, 81.5% (2793 transcripts, 2377 genes) of the transcripts were assigned to 1615 contigs of the recently reported intersect transcriptome (from bryozoan and rainbow trout, Faber *et al*., 2021). A total of 634 transcripts (528 genes), did not have a hit within the intersect transcriptome and are hence unique to our assembly (Supplementary Fig. 6A). In comparison of the transcripts that matched to the Faber *et al*., 2021 transcriptome, the unique transcripts had higher relative proportion of genes related to Ribosome (5.13%), Ribosome biogenesis (3%), Ubiquitin system (2%) and Peptidases and inhibitors (1.6%) based on BRITE hierarchies (Supplementary Fig. 6B). GO terms comparison using WEGO 2 showed that the unique *T. bryosalmonae* transcripts have higher (*P* < 0.05) proportion of genes related to the cellular component terms: Endoplasmic reticulum membrane (GO:0005789) and Mitochondrial protein complex (GO:0098798), and following molecular function terms: Structural molecule activity (GO:0005198) and Structural component of ribosome (GO:0003735) (Supplementary Fig. 6C and D).

### Differentially expressed *T. bryosalmonae* genes linked to PL

**Fig. 4.**
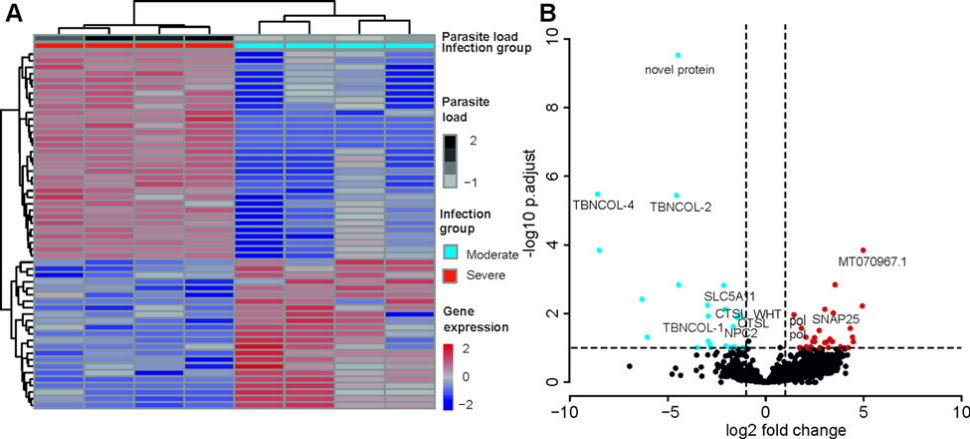
Differential transcript expression and GO enrichment: (A) Heatmap showing variance stabilized expression of the differentially expressed transcripts between the moderately and severely infected hosts. (B) Volcano plot showing the fold-change differences between the moderately and severely infected hosts. Light blue and red points represent the down- and up-regulated genes in severely infected fish, respectively.

A total of 55 *T. bryosalmonae* genes were differentially expressed at FDR <0.1 between severely and moderately infected fish (Fig. 4; Supplementary Table 9). Similar to the whole transcriptome annotation, only a proportion of differentially expressed genes (*n* = 31) retrieved functional annotation using KAAS and Trinotate. In the severely infected samples, the polar capsule related minicollagens (TBNCOL-1, TBNCOL-2 and TBNCOL-4) were downregulated, indicating that severely and moderately infected hosts may differ in the extent of polar capsule formation of *T. bryosalmonae*. Furthermore, in severely infected fish several lysosome and lipid metabolism-related genes were downregulated. These include cathepsin L (CTSL; 2 genes), Niemann-Pick C2 protein (NPC2), hydroxymethylglutaryl-CoA synthase (HMGCS), hydroxymethylglutaryl-CoA reductase (NADPH) (HMGCR) and PI-PLC X domain-containing protein 2 (PLCX2). Interestingly, differential expression of CTSLs has recently been found between the proliferative blood- and sporogonic gill stages of the myxozoan *S. molnari* (Hartigan *et al*., 2020). Therefore, it is possible that cathepsin L is also linked to sporogony in *T. bryosalmonae*. In severely infected fish downregulation was also inferred for the expression of glutathione peroxidase (gpx; the highest mean expression level among differentially expressed genes; AvgFPKM in the Supplementary Table 9), which is an antioxidant that protects the parasite from the damage caused by free radicals released from the host immune cells (Changklungmoa *et al*., 2018).

Among the upregulated genes in the severely infected fish, four were retrotransposon genes (annotated as Retrovirus-related Pol polyprotein from type-1 retrotransposable element R2; pol). The upregulated gene with the highest fold change corresponded to low-density lipoprotein receptor class A-like protein 3 (MT070967.1, 100% BLASTN identity) gene. Other upregulated genes included Talin (TLN), TRAF2 and NCK interacting kinase (TNIK), Tetraspanin-9 (TSN9), Vacuolar-sorting protein (snf7), importin-5 (IPO5), calcium-dependent secretion activator (CADPS), Rho GTPase-activating protein 39 (ARHGAP39), synaptosomal-associated protein 25 (SNAP25), AGPAT8 lysocardiolipin and lysophospholipid acyltransferase (LCLAT1) and large subunit ribosomal protein L3e (RP-L3e).

### Drug targets and homology modelling

**Fig. 5.**
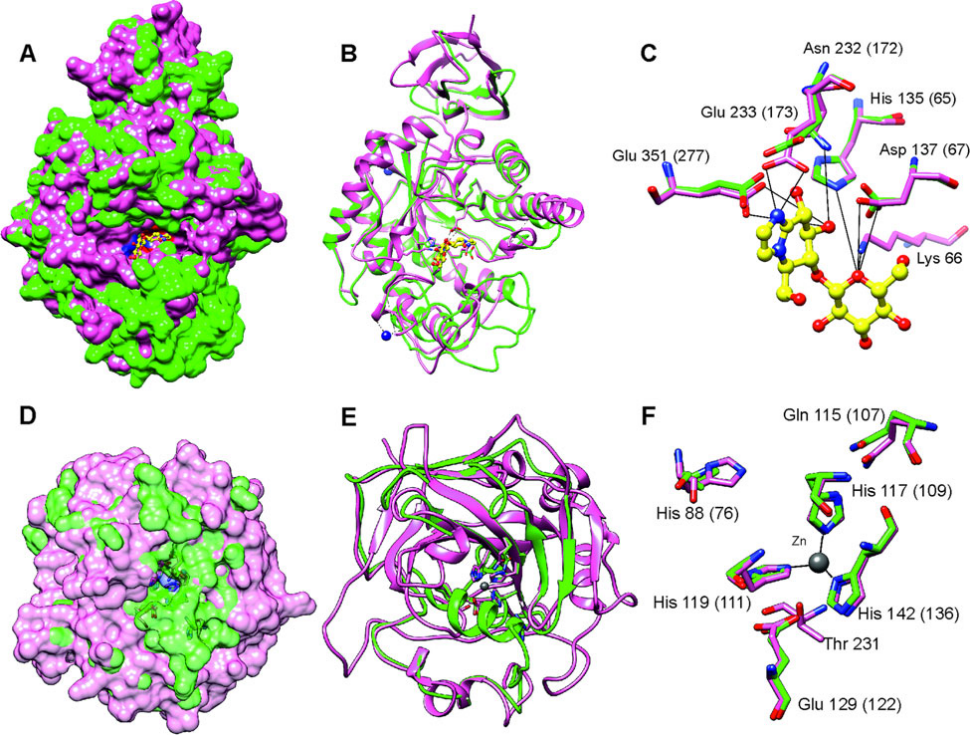
Prediction of the EGCase2 (DN1345_c0_g1_i1) and CA2 (DN419_c0_g2_i1) three-dimensional structures in *T. bryosalmonae*. (pink). (A) The parasite EGCase2 (green) structure is superimposed on the chain B of the template endoglycoceramidase ii from *rhodococcus sp.* In EGCase2, the inhibitor (yellow) is shown in ball and stick representation. Similar binding cavities between template and target can be seen around the inhibitor molecule. (B) The same structure shown in ribbons to illustrate the consistency between the template and target structures. (C) The interactions between the EGCase II amino acids and inhibitor at the binding site are shown with black lines as reported in Caines *et al*. (2007). (D) The parasite CA2 (green) structure is superimposed on the chain A of the *Schistosoma mansoni* carbonic anhydrase (SmCA, pink). The surface is made transparent to show the active site. (E) The same structure shown in ribbons to illustrate the consistency between SmCA and the predicted *T. bryosalmonae* structures. (F) The active site (His^76^, Gln^107^, Glu^122^, and Thr missing) and Zinc binding Histidines (His^109^, His^111^ and His^1136^) residues of *T. bryosalmonae* CA2 also acquired similar orientation as the SmCA (Da'dara *et al*., 2019).

After stringent screening of 3427 putative parasite transcripts against known drug target proteins in the ChEMBL database, four potential chemotherapeutic targets, Endoglycoceramidase (two genes; EGCase1 and EGCase2), Legumain-like protease, Carbonic anhydrase 2 and Pancreatic lipase-related protein 2 (two genes) were identified (Table 2). The two EGCase transcripts encode 283 and 382 amino acid long partial protein sequences with 43.8% similarity. We subsequently individually modelled the 3D structures of the two EGCase and CA2 protein sequences by using Phyre2. For both EGCase1 and EGCase2, >90% of residues were modelled at >90% confidence during homology modelling. Both structures showed a high overlap with the template structure (Fig. 5, Supplementary Fig. 7 and 8). The structure of the EGCase2 (longer alignment length with the template) is shown in Fig. 5 in which the low confidence residues (92–114) were unreliably modelled into a loop because they were missing in the template sequence (Supplementary Fig. 8). The template EGCase chosen by Phyre2 (B chain of endoglycoceramidase ii from *Rhodococcus* sp. (PDB ID 2OYL) for the homology modelling was bound to an inhibitor molecule (cellobiose-like imidazole) using polar interactions with Lys^66^, His^135^, Asp^137^, Asn^232^, Glu^233^ and Glu^351^ (Caines *et al*., 2007). Apart from Lys^66^, which was missing from the partial template sequence, the corresponding His^65^, Asp^67^, Asn^172^, Glu^173^ and Glu^277^ in the *T. bryosalmonae* EGCase2 (DN1345_c0_g1_i1, Fig. 5c) acquired the same positions in the binding site (Fig. 5C). The *T. bryosalmonae* EGCase1 (DN11176_c0_g2_i1) exhibits three amino acids occupying the similar 3D confirmation as the template structure's binding site amino acids (Supplementary Fig 7C). These conserved binding site residues of *T. bryosalmonae* Endoglycoceramidase II represent a promising target for the chemotherapeutic drugs with cellobiose-like imidazole inhibitor-like structures.

**Table 2.** Potential drug targets of the *de novo* assembled *T. bryosalmonae* transcripts

Target protein (abbreviation; CHEMBL ID) (original publication)	Role in metabolism	Drug target in other parasites	Potential inhibitors from literature	Transcript ID	Average FPKM	*E*-value	Identity (%)	Alignment length (aa)
Endoglycoceramidase (EGCase; CHEMBL3112388) (Ishida *et al.,* 2014)	Lipid digestion	None	Cellobiose-like imidazole and iminocyclitol inhibitors (Caines *et al.,* 2007)	DN11176_c0_g2_i1 (EGCase1)	185.3	6.97×10^−9^	28.8	139
				DN1345_c0_g1_i1 (EGCase2)	152.5	1.98×10^−8^	25.6	160
Legumain-like protease (LGMN; CHEMBL1075261) (Ovat *et al.,* 2009)	Proteolytic (lysosomal) enzyme	*Schistosoma mansoni* (Ovat *et al.*, 2009) and *Trichomonas vaginalis* (Rendón-Gandarilla *et al.*, 2013)	Aza-Peptidyl Michael Acceptor and Epoxide selective inhibitors (Ovat *et al.,* 2009).	DN3668_c0_g1_i1	193.5	2.86×10^−6^	24.5	139
Carbonic anhydrase 2 (CA2; CHEMBL2331045) (Syrjänen *et al.,* 2013)	Reversible hydration of carbon dioxide	*Trypanosoma* and *Leishmania* (protists) (Vermelho *et al.*, 2017) and blood flukes, Schistosomes (Angeli *et al.*, 2020)	Sulfonamide, thiol and hydroxamate inhibitors (Vermelho *et al,.* 2017; Angeli *et al.*, 2020)	DN419_c0_g2_i1	105.2	5.29×10^−5^	31.6	114
Pancreatic lipase-related protein 2 (PLRP2; CHEMBL2169729) (Point *et al.,* 2012)	Lipid digestion	None but its inhibitor Orlistat has shown *in vitro* growth inhibition of a protist parasite *Giardia duodenalis* (Hahn *et al.*, 2013)	Orlistat (lipase inhibitor) and Oxadiazolones (Point *et al.,* 2012)	DN10075_c0_g2_i1	92.0	0.000355	26.8	112
				DN9655_c0_g2_i1	74.9	0.000418	22.2	149

More than 90% of residues of the *T. bryosalmonae* CA2 were modelled at >90% confidence using Phyre2. The predicted structure (Fig. 5D and E, green) was superimposed on chain A of the parasite *Schistosoma mansoni* carbonic anhydrase (SmCA, PDB ID 6QQM, pink). In SmCA, the active site residues are His^88^, Gln^115^, Glu^129^, and Thr^231^ and the Zinc binding Histidines are His^117^, His^119^ and His^142^ (Da'dara *et al*., 2019). These important residues are also conserved in *T. bryosalmonae* CA2 (Active site: His^76^, Gln^107^, Glu^122^ and Zinc binding Histidines: His^109^, His^111^ and His^136^) while only Threonine is missing. These residues also acquired similar orientation as in the SmCA (Fig. 5F) thus the inhibitors used for the *S. mansoni* carbonic anhydrase might also be effective for *T. bryosalmonae* CA2. Finally, we did not predict 3D structures of LGMN and PLRP2s as they are partial sequences and lacked important active site or binding site residues based on alignments with ChEMBL hits (Supplementary Fig. 9 and 10).

## Discussion

This study reports the first annotated transcriptome of *T. bryosalmonae* derived from wild, infected brown trout using dual RNA sequencing. By employing multi-step filtering of parasite reads followed by *de novo* assembly, our work provides new insights into the differentially expressed *T. bryosalmonae* genes in the fish and identifies six proteins that could act as potential targets for the development of antiparasitic drugs against PKD. As such, this work demonstrates the utility of the assembled *T. bryosalmonae* transcriptome and promotes experimental approaches to develop and test new control strategies against PKD.

A major challenge for the *de novo* transcriptome assembly using dual-RNA sequencing data is to correctly separate host and parasite RNA reads (Schulze *et al*., 2016; Videvall *et al*., 2017). In earlier studies targeting myxozoan transcriptomes within fish hosts, RNA was isolated from the appendages visible by eye, e.g. plasmodia from the *K. iwatai* infected gilthead seabream (Chang *et al*., 2015) or cysts from the *T. kitauei* infected common carp (Yang *et al*., 2014), and after careful washing steps that were followed by *in silico* filtering of reads to avoid inclusion of host sequences during the parasite transcriptome or genome assembly. Yet, more recent works have shown that host tissue contamination is still an important issue in next generation sequencing of myxozoans (Foox *et al*., 2015; Alama-Bermejo *et al*., 2020; Hartigan *et al*., 2020). Furthermore, isolation of microscopic *T. bryosalmonae* specimens from salmonid kidney tissue is challenging and our initial RNAseq data was dominated (more than 97% of total sequences) by brown trout RNA reads. Therefore, in order to produce a host-free parasite transcriptome, we employed a rigorous multi-step strategy, comprising host genome mapping, pre-assembly metagenomic classification and post-assembly filtering. Recently, Alama-Bermejo *et al*. (2020) tackled the problem of host contamination removal from the myxozoan *C. shasta* transcriptome by employing a two-step filtering approach. In the first step, host reads were removed by aligning reads to host and parasite reference genomes. During the second step (transcript-level filtering), Alama-Bermejo *et al*. (2020) used BLASTN against both host and parasite genomes to further exclude the host transcripts, while contaminant sequences (of bacterial, fungal or viral origins) were excluded based on BLASTX (Alama-Bermejo *et al*., 2020). In contrast, *T. bryosalmonae* transcriptome was not published at the time of the analysis. In addition, only a good quality genome of *Salmo salar*, representing a genetically close sister species to brown trout, was available for us at the time of the analysis. Therefore, to overcome these limitations, we included multiple myxozoan genomes in the Kraken2 database to classify the unmapped reads and assembled parasite transcriptome using reads that were not classified to ‘bony fishes’. Furthermore, reads from the uninfected fish that mapped on our assembled transcripts provided a useful control step which enabled to effectively remove both known and unknown transcripts originating from the fish host. Some homology between host and parasite short reads is expected and therefore we allowed up to 10% of the total reads per transcript to map to the uninfected fish. Similar to the second step transcript-level filtering in *C. shasta* (Alama-Bermejo *et al*., 2020), we also excluded transcripts that showed significant (e.g. bacterial, viral and vertebrate) BLAST/Kraken2 hits during the post-assembly filtering, thus any potential bacterial and human contamination during the sample collection and library preparation was also filtered out. Finally, the majority of transcripts with positive correlation between raw read counts and PL measured by an independent approach (qPCR; Bruneaux *et al*., 2016; Debes *et al*., 2017) indicated that our pipeline successfully removed the host sequences and captured true RNA reads from *T. bryosalmonae*.

Myxozoans are highly reduced metazoans with extremely small diverged genome sizes (22.5–188.5 Mb), which makes it challenging to evaluate the completeness of assembled transcriptomes and genomes (Chang *et al*., 2015; Hartigan *et al*., 2020; Yahalomi *et al*., 2020). By using a large set of published data, we identified a set of 231 BUSCO genes that were detected in all myxozoans. Based on this smaller set of BUSCO genes, the estimated *T. bryosalmonae* transcriptome assembly completeness is close to 56.3% (130 of 231). Thus, we suggest that this core set of 231 BUSCO genes can be used as an alternative to estimate the completeness of the assemblies in future myxozoan studies. Conversely, we also observed that 23.7% of metazoan single-copy gene genes were not identified in any published myxozoan genome and transcriptome assemblies. As the myxozoans have gone through an extreme reduction in body plan and genome size (Chang *et al*., 2015), it is possible that at least some of these genes have been truly lost during myxozoan evolution. However, we cannot exclude, at this point, the alternative explanation that unidentified BUSCO genes are still present in myxozoans, but because of highly divergent nature, were not detected using BUSCO.

Currently, there are no licenced drugs available for efficient PKD treatment. Earlier studies demonstrated an efficiency of malachite green (triarylmethane dye; Clifton-Hadley and Alderman, 1987) and fumagillin (antibiotic of the fungus *Aspergillus fumigatus*; Le Gouvello *et al*., 1999) for treatment against PKD. Malachite green has been used for many fish species to control fungal attacks, protist infections and other diseases, such as caused by helminths (Srivastava *et al*., 2004). Fumagillin has been shown to prevent clinical outbreaks of whirling disease caused by the myxosporean parasite *Myxobolus cerebralis* in rainbow trout (El-Matbouli and Hoffmann, 1991). In addition, fumagillin is an effective antibiotic against the microsporidian pathogen *Nosema apis* in honey bees (Burnham, 2019). However, because of the accumulation of malachite green in fish and its potential teratogenic and carcinogenic properties, its use is, unsurprisingly, restricted for food fish. Similarly, it has been shown that fumagillin treatment leads to a dramatic loss in haematopoietic tissue in rainbow trout (Wishkovsky *et al*., 1990). Therefore, given the high economic losses incurred by PKD on the freshwater salmonid aquaculture industry of Europe and North America (Feist, 2004; Burkhardt-Holm *et al*., 2005; Borsuk *et al*., 2006; Okamura *et al*., 2011), novel treatment targets and compounds are urgently needed.

The assembly of the *T. bryosalmonae* transcriptome enabled us to identify potential drug targets while avoiding potential toxicity to the salmonid host due to drug target homology. The top drug candidate (based on the lowest BLAST E-value) endoglycoceramidase (EGCase) is an enzyme that hydrolyses acidic and neutral glycosphingolipids to oligosaccharides and ceramides. Our homology modelling analysis predicted that the *T. bryosalmonae* EGCase is expected to have the similar binding site as the original template and hence the same inhibitors, such as iminocyclitols, may affect this enzyme activity in *T. bryosalmonae*. Among the free-living Cnidaria, EGCase has been previously reported to occur in *Cyanea nozakii* (Jellyfish) (Horibata *et al*., 2000) and *Hydra vulgaris* (Hydrozoa) (Horibata *et al*., 2004). Interestingly, EGCase is involved in a unique dietary glycosphingolipids catabolism pathway in *Hydra* (Horibata *et al*., 2004). However, until this study, endoglycoceramidases have not been reported in Myxoza. The presence of two copies of this gene in a reduced cnidarian genome makes it possible that the glycosphingolipids catabolism pathway also plays a role in the parasite lipid metabolism.

The second identified drug target legumain-like protease, an asparaginyl endopeptidase, occurs in the gut of the hard tick (*Ixodes ricinus*) where it participates in the digestion of blood meal haemoglobin (Sojka *et al*., 2007). A similar legumain enzyme is also present in the parasitic blood fluke *Schistosoma mansoni* (Davis *et al*., 1987). The proteolytic activity of legumain can be reduced by Aza-peptidyl Michael acceptors, which are inhibitors for the legumains (Götz *et al*., 2008; Ovat *et al*., 2009; Rendón-Gandarilla *et al*., 2013). For example, Aza-peptidyl Michael acceptors inhibit legumain activity in the living cells of protist *Trichomonas vaginalis* and reduce the parasitic cytoadherence to the host cells (Rendón-Gandarilla *et al*., 2013). Similarly, Aza-peptidyl Michael acceptors (Götz *et al*., 2008) and epoxide inhibitors (Ovat *et al*., 2009) have been shown to inhibit legumain in other evolutionary distant parasite species, such as *S. mansoni* and *I. ricinus*.

The third drug target, Carbonic anhydrase 2 (CA2), belongs to the plasma membrane anchored alpha carbonic anhydrases, which catalyse the reversible hydration of carbon dioxide and aid in intracellular pH regulation. Carbonic anhydrases are considered as potent anti-parasitic drug targets against protists, such as *Trypanosoma*, *Leishmania* (Vermelho *et al*., 2017) and, recently, against blood flukes, schistosomes (Da'dara *et al*., 2019; Angeli *et al*., 2020). In the predicted three-dimensional model, the active site- and Zn binding residues of *T. bryosalmonae* CA2 retained similar positions as in SmCA, suggesting that similar therapeutic inhibitors may be used to target this protein in *T. bryosalmonae*. The schistosome alpha carbonic anhydrase plays a role in parasite virulence as its suppression using RNAi significantly reduced the establishment of parasite within host (Da'dara *et al*., 2019). Several sulphonamide, thiol and hydroxamate substances have been shown to inhibit CA2 in *Trypanosoma* and *Leishmania* schistosomes (Vermelho *et al*., 2017; Angeli *et al*., 2020).

The fourth drug target, pancreatic lipase-related protein 2 (PLRP2), is a secretory lipase enzyme that in vertebrates hydrolyses both phospholipids and galactolipids and helps in milk fat digestion (Berton *et al*., 2009). This gene is also present in genomes of several cnidarians, including *Exaiptasia pallida* (NCBI Gene IDs: 110254178 & 110231612), *Stylophora pistillata* (NCBI Gene IDs: 111321164 & 110253264) and *Nematostella vectensis*, (NCBI Gene IDs: 5496642). Presence of PLRP2 in invertebrates and cnidarians suggest a potential role in nutrient acquisition and lipid metabolism. In humans, this enzyme has been employed as a target for the lipase inhibitors, such as Orlistat, for obesity treatment (Ballinger and Peikin, 2002). Interestingly, Orlistat has also been shown to inhibit *in vitro* growth of a protist parasite *Giardia duodenalis* (Hahn *et al*., 2013). Altogether, earlier work on endoglycoceramidases, legumain-like proteases, carbonic anhydrases and lipase inhibitors suggests that the identified potential anti-parasitic drug targets represent promising chemotherapeutic candidates also against myxozoan *T. bryosalmonae.*

The pathways and enzymes assigned by the KEGG orthology provided important functional information about the genetic make-up of *T. bryosalmonae*. Among identified pathways, the endocytosis pathway is expected to have an important role in *T. bryosalmonae.* Specifically, endocytosis is involved in acquiring nutrients in other Myxozoa (Yang *et al*., 2014). In addition, endocytosis of the cellular content of the host phagocyte (that has already engulfed the parasite cell) has been observed in *T. bryosalmonae* (Morris and Adams, 2008). Endocytosis has also been observed during the transfer of material from the primary cell to the enclosed secondary and tertiary cells in *T. bryosalmonae* (Morris and Adams, 2008), suggesting that endocytosis may have wider functions than acquiring nutrients. Furthermore, it has been shown that the internal cells (both secondary and tertiary) of *T. bryosalmonae* are formed *via* the engulfment of other cells by primary cell (Morris, 2010) and thus, the same molecular machinery i.e. endocytosis pathway genes are likely to be involved in these processes.

Peptidases (proteases) were the second most abundant enzymes found *T. bryosalmonae.* These are important catabolic enzymes that hydrolyze proteins into smaller polypeptides or single amino acids. A key role of the proteases in the nutrient digestion has already been proposed for the myxozoan *T. kitauei* (Yang *et al*., 2014). Furthermore, proteases are known virulence factors in many parasites and considered as important targets for the antiphrastic drug development (McKerrow *et al*., 2006; Pina-Vazquez *et al*., 2012; Siqueira-Neto *et al*., 2018). More recently, proteases have also been suggested as potential drug targets in myxozoan *S. molnari* (Hartigan *et al*., 2020). In the current *T. bryosalmonae* assembly, the distribution of KEGG assigned proteases showed higher abundance of cysteine (32%) and metallo (27%) proteases, while a smaller proportion of serine (16%), threonine (16%) and aspartic proteases (8%) were identified (Fig. 3C). In contrast, recently published *T. bryosalmonae* assembly (Faber *et al*., 2021) reported a slightly higher proportion of metallo proteases in relation to cysteine and serine proteases. This difference may reflect the host-specificity of proteases as the former transcriptome was common to both rainbow trout and bryozoan host, while the latter was based on a native salmonid host, brown trout. Furthermore, despite low completeness of current transcriptome assembly, the protease composition of *T. bryosalmonae* was very similar to *S. molnari*, *C. shasta*, *K. iwatai* and *M. cerebralis*. Noteably, *T. kitauei* exhibited a clearly different protease composition compared to other myxozoans (Fig. 3C).

Between severely and moderately infected fish, altogether, 55 *T. bryosalmonae* genes were differentially expressed. Most interestingly, a higher expression of minicollagens, which are expected to be only expressed during myxozoan sporogenesis, in moderately infected fish suggests that more parasites had reached the sporogonic stage in moderately infected relative to the severely infected fish. Similarly, Niemann-Pick C2 protein and ammonium transporter Rh, whose homologs are found in the polar capsule of the myxozoan *C. shasta* (ecdysteroid-regulated 16 kDa-like and ammonium transporter Rh type C 2-like respectively) (Piriatinskiy *et al*., 2017) were upregulated in moderately infected fish. Thus, it is possible that fish with low PL shows increased abundance of sporogonic stages of *T. bryosalmonae.* However, further experimental time-series analysis is needed to confirm this hypothesis. Among the genes upregulated in severely infected fish was Talin, which is related to the cytoskeletal adhesion to the extracellular matrix (Klapholz and Brown, 2017). Recently, the upregulation of the Talin gene in *C. shasta* has been shown to associate with the transformation from a localized (intestine) to a proliferated infection of other organs in rainbow trout (Alama-Bermejo *et al*., 2019). Thus, Talin, as a putative virulence factor, may also play role in proliferation and adhesion in *T. bryosalmonae*.

Based on the 231 metazoan genes detected in all published myxozoan transcriptomes/genomes using BUSCO, we estimated that the assembled transcriptome recovered approximately ~56% of *T. bryosalmonae* genes (~92% for intersect transcriptome of Faber *et al*., 2021). The removal of transcripts with low expression levels (less than 40 reads), is one likely reason behind the low BUSCO score and genes with low expression levels are most likely underrepresented in the current transcriptome assembly. Secondly, mapping of the reads against multiple fish genomes is expected to efficiently exclude teleost sequences but it also removes parasite reads homologous to the host (Schulze *et al*., 2016). This, however, reduces potential toxicity due to nucleotide level homology between the host and parasite and facilitates drug target discovery process. Thirdly, RNA sequencing has known bias against extremes of GC content (Swindell *et al*., 2014; Sheng *et al*., 2016) and the AT-richness of the *T. bryosalmonae* transcripts may have resulted in an underrepresentation of the parasite reads in the raw data. This might be one potential reason why only ~10% protein sequences in the current *T. bryosalmonae* transcriptome assembly are complete (i.e. having start and stop codons at the opposite ends). In addition, our filtering pipeline most likely removed a proportion of the true parasite reads from the further assembly, reflecting the trade-off between false positive and negative identification of parasite reads in dual-RNA sequencing experiments. Finally, despite applying two different strategies for the transcriptome annotation, one fourth (24.6%) of the transcripts still remained uncharacterized. This demonstrates the urgent need for expanding myxozoan genomic and proteomic resources. Despite the limitations, this study illustrates the value and utility of our *T. bryosalmonae* transcriptome assembly by enabling the identification of novel drug targets providing new biological insights into both parasite biology and disease progression.

In conclusion, we developed an *in silico* pipeline to efficiently separate transcripts of *T. bryosalmonae* from the kidney tissue of a native fish host, followed by *de novo* transcriptome assembly. As such, this study represents an important milestone towards developing new effective control strategies against PKD. More generally, we expect that the transcriptome assembly of *T. bryosalmonae* represents a valuable resource for future studies on genomic architecture and evolution of the myxozoans and will facilitate research on molecular mechanisms underpinning PKD.

## Supplementary material

For supplementary material accompanying this paper visit https://doi.org/10.1017/S003118202100010X. click here to view supplementary material
