# Supplementary material for: Know your enemy – transcriptome of myxozoan Tetracapsuloides bryosalmonae reveals potential drug targets against proliferative kidney disease in salmonids
Source: Parasitology. 2021 Jan 22;148(6):726–39. doi: 10.1017/S003118202100010X (PMC8056827; doi:10.1017/S003118202100010X)
Supplement: Supplementary file 1 [file S003118202100010Xsup.zip › S003118202100010Xsup002.docx]

**Appendix, supplementary information**

**Know your enemy - transcriptome of myxozoan *Tetracapsuloides bryosalmonae* reveals potential drug targets against proliferative kidney disease in salmonids**

Freed Ahmad^1^*, Paul V. Debes^2^, Lilian Pukk^3^, Siim Kahar^3^, Hanna Hartikainen^4^, Riho Gross^3^, Anti Vasemägi^3,5^

^1^Department of Biology, University of Turku, FI-20014, Finland;

^2^ Department of Aquaculture and Fish Biology, Hólar University, Saudárkrókur, Iceland;

^3^Chair of Aquaculture, Institute of Veterinary Medicine and Animal Sciences, Estonian University of Life Sciences, 51014 Tartu, Estonia;

^4^School of Life Sciences, The University of Nottingham, University Park, NG7 5RD, Nottingham, UK; and

^5^Department of Aquatic Resources, Swedish University of Agricultural Sciences, 17893 Drottningholm, Sweden.

**Running title:** *Tetracapsuloides bryosalmonae* transcriptome assembly

^*^Correspondence to:

Anti Vasemägi

^5^Department of Aquatic Resources, Swedish University of Agricultural Sciences, 17893 Drottningholm, Sweden; ^3^Chair of Aquaculture, Institute of Veterinary Medicine and Animal Sciences, Estonian University of Life Sciences, 51014 Tartu, Estonia

Anti.vasemagi@slu.se


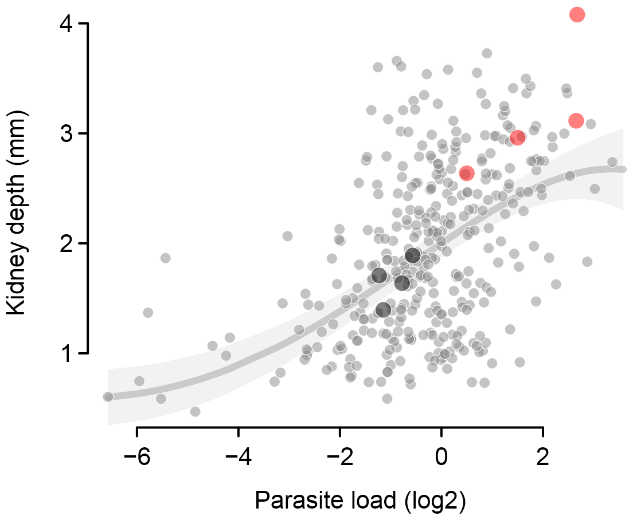


**Supplementary figure 1.** Relative parasite load vs. length-standardized kidney depth in all infected sampled fish sampled (dots) in the River Mustoja in 2015 and the fitted overall second-order polynomial regression line with 95% confidence bands as in Debes *et al.* (2017). The enlarged dots highlight the moderately (black; n = 4) and severely infected (red; n = 4) fish as used in transcriptomic analyses.


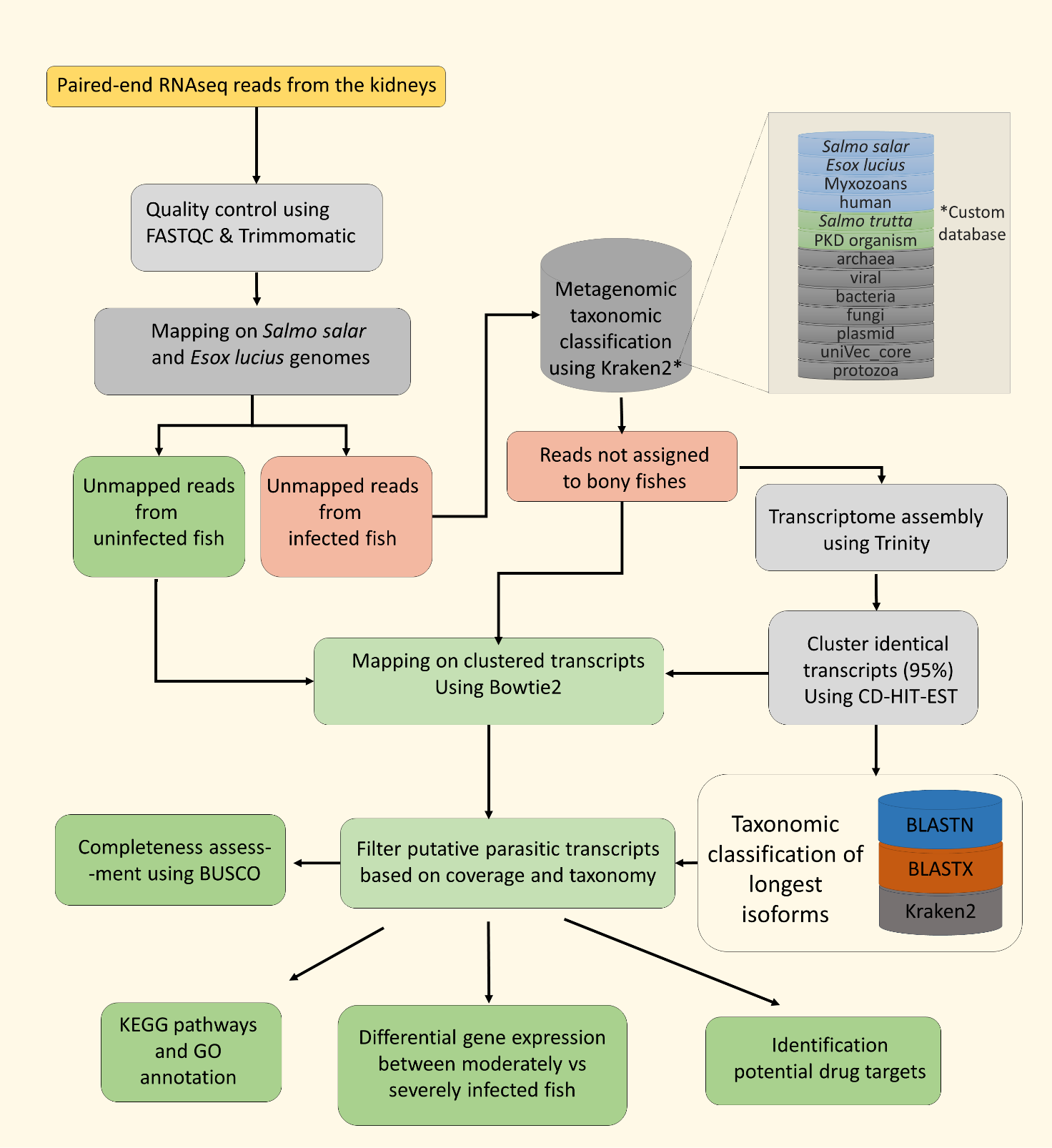


**Supplementary figure 2.** Data analysis pipeline.


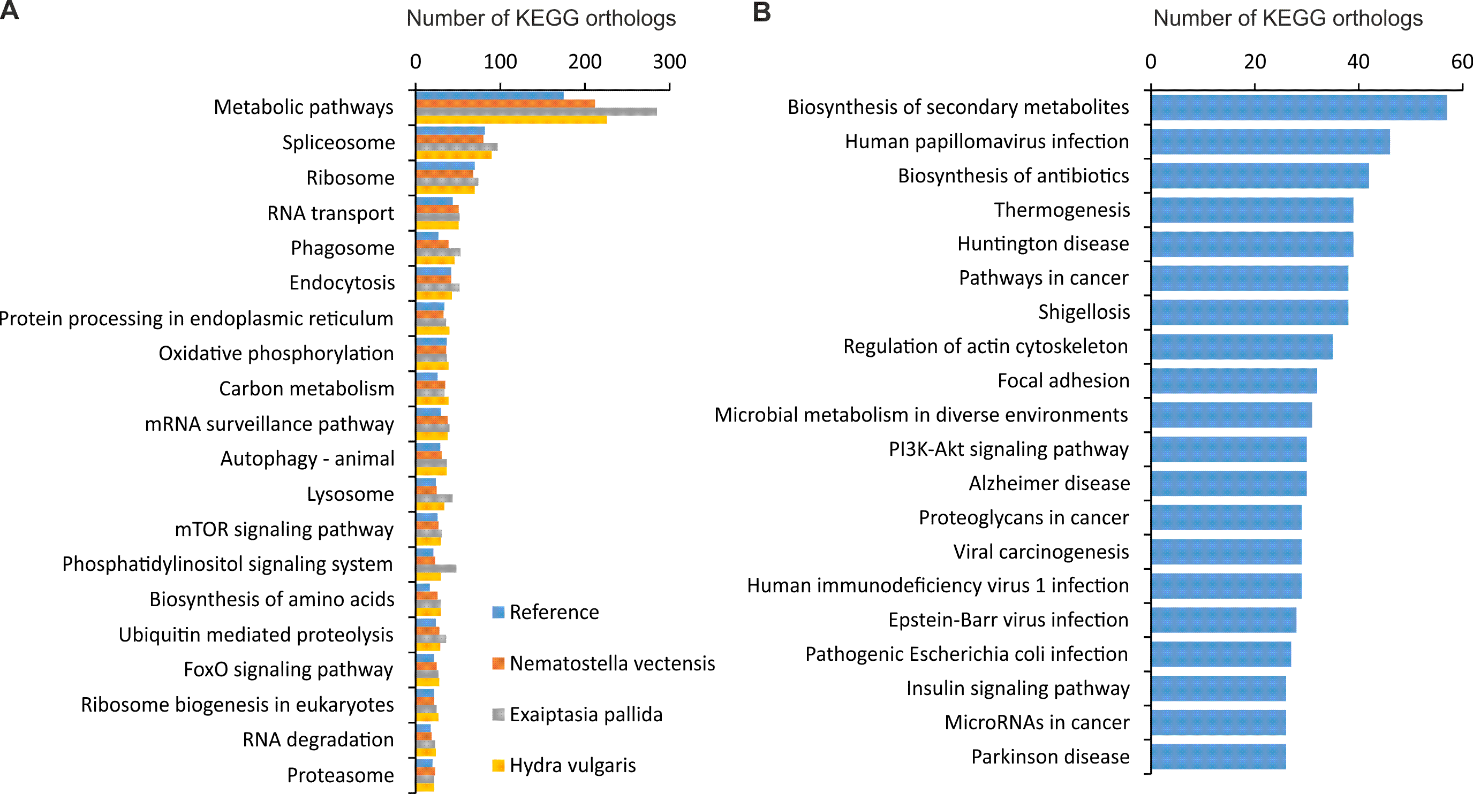


**Supplementary figure 34.** Top KEGG pathways in the myxozoan parasite transcriptome **A**) Top 20 pathways found in both reference and cnidarian-specific searches **B**) Top 20 pathways found only in reference KEGG orthology.


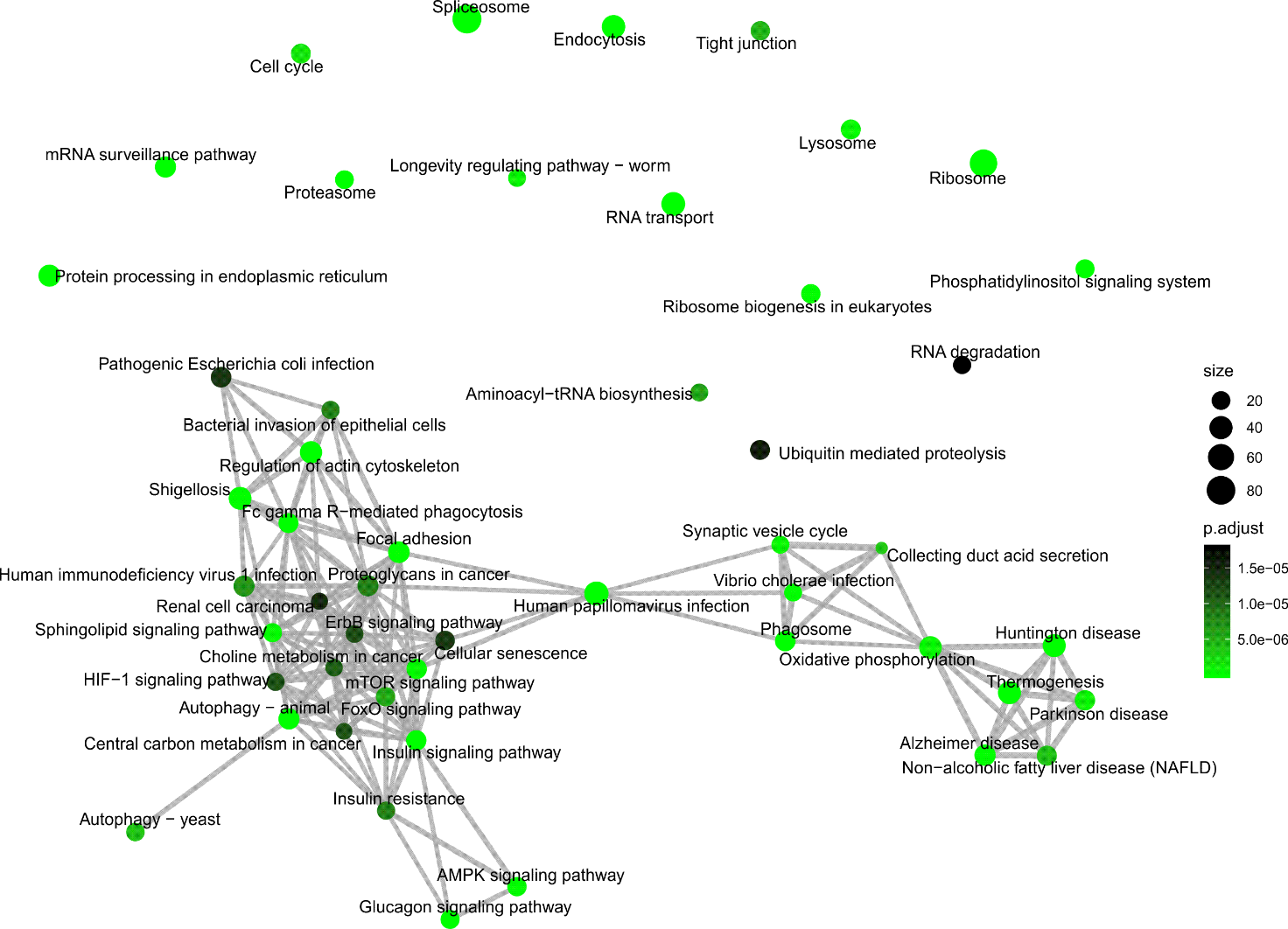


**Supplementary figure 45.** Top 50 enriched KEGG pathways as enrichment network map. The overlapping genes in the enriched pathways are shown as edges with mutually overlapping gene sets clustering together forming functional module.

**
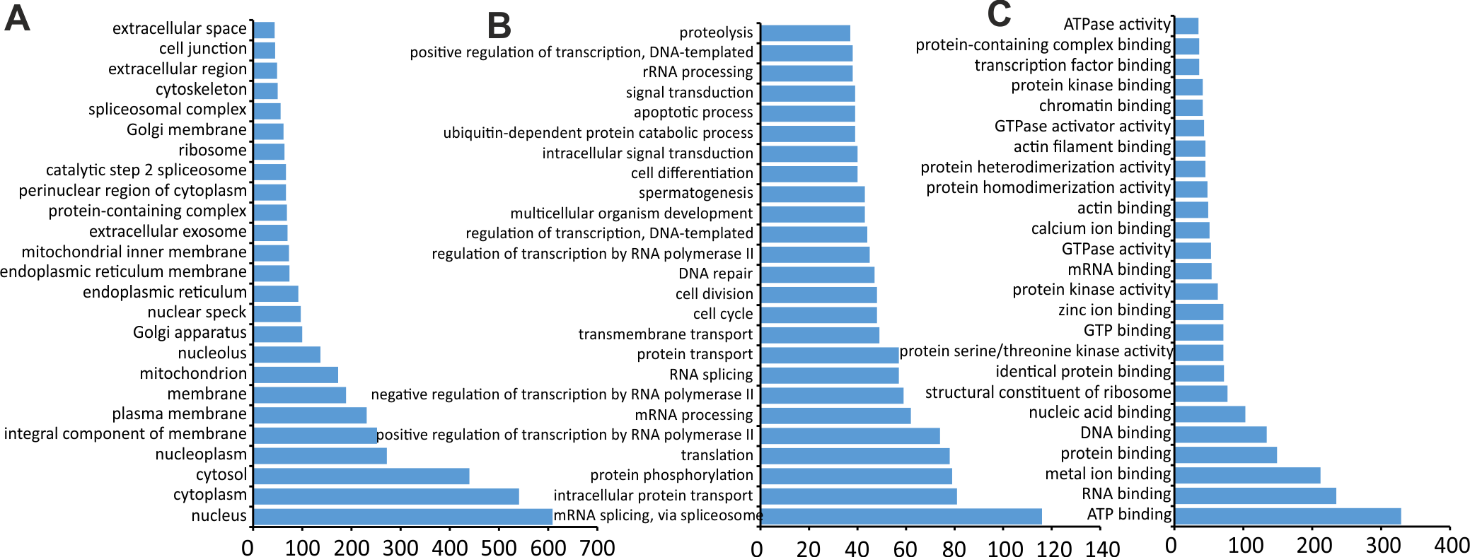
 Supplementary figure 56.** Top 25 most abundant GO terms in **A**) Cellular component **B**) Biological process and **C**) Molecular function.


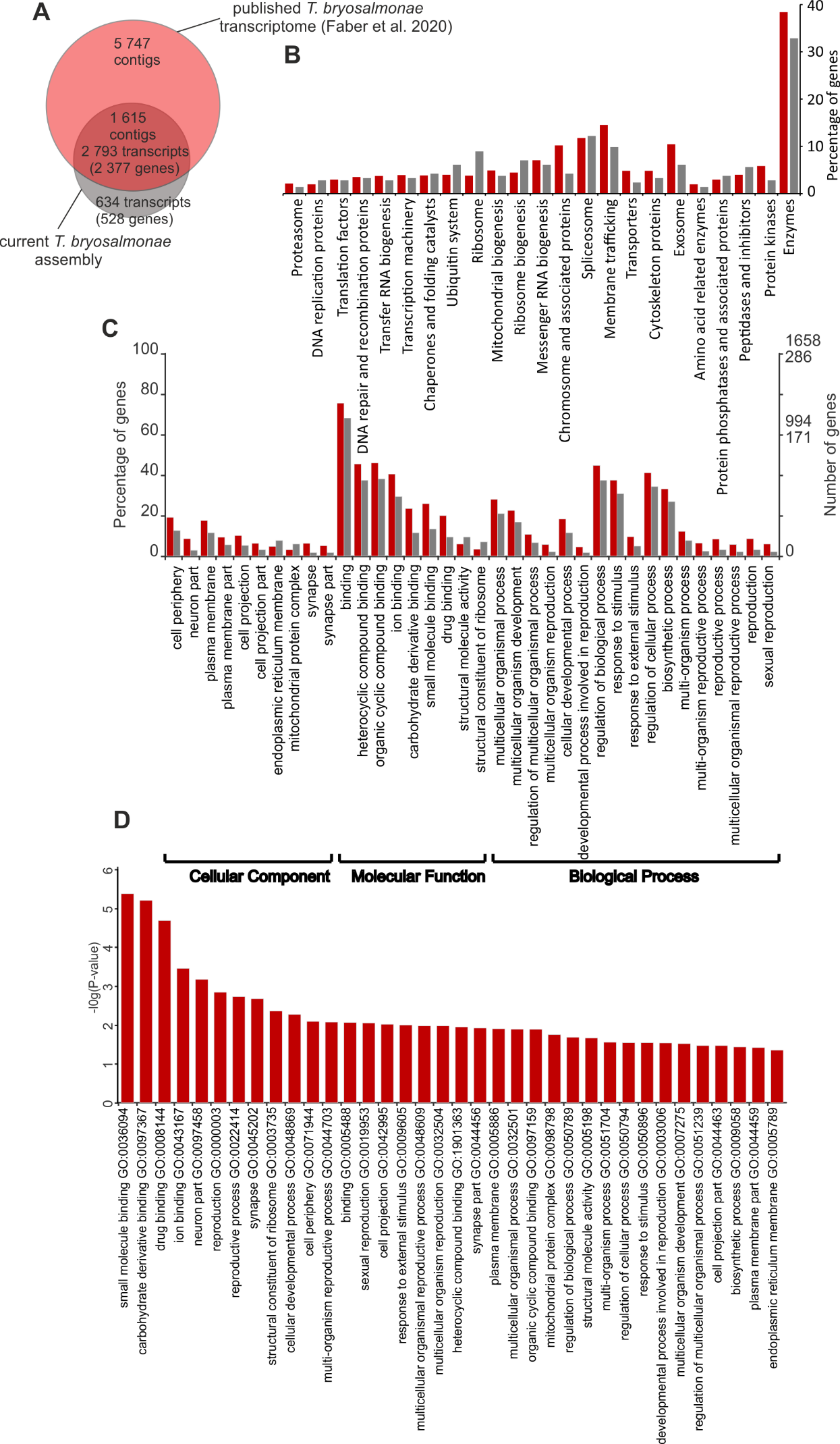


**Supplementary figure 6.** Overlap between our *T. bryosalmonae* assembly and intersect transcriptome of Faber *et al.* (2020). **A)** Venn diagram showing overlap between two assemblies using reciprocal BLASTN. Pink circle represents genes present in the intersect transcriptome (Faber *et al.* 2020) and grey circle corresponds to *T. bryosalmonae* transcripts identified in current work. The overlap between two circles represents genes found in both assemblies. **B)** Percentage of genes in different KEGG Brite hierarchies for the genes that were present in both assemblies (red) and the genes present in only current assembly. **C)** Trinotate assigned GO term differences between the genes that were found in the intersect transcriptome (Faber *et al*. 2020) and the genes that were unique to our study. Only genes having at least one GO term were used as inputs for both lists. **D)** Significantly different GO terms are shown in terms of –log (P-value). Both **C** & **D** are generated using (WEGO 2.0).


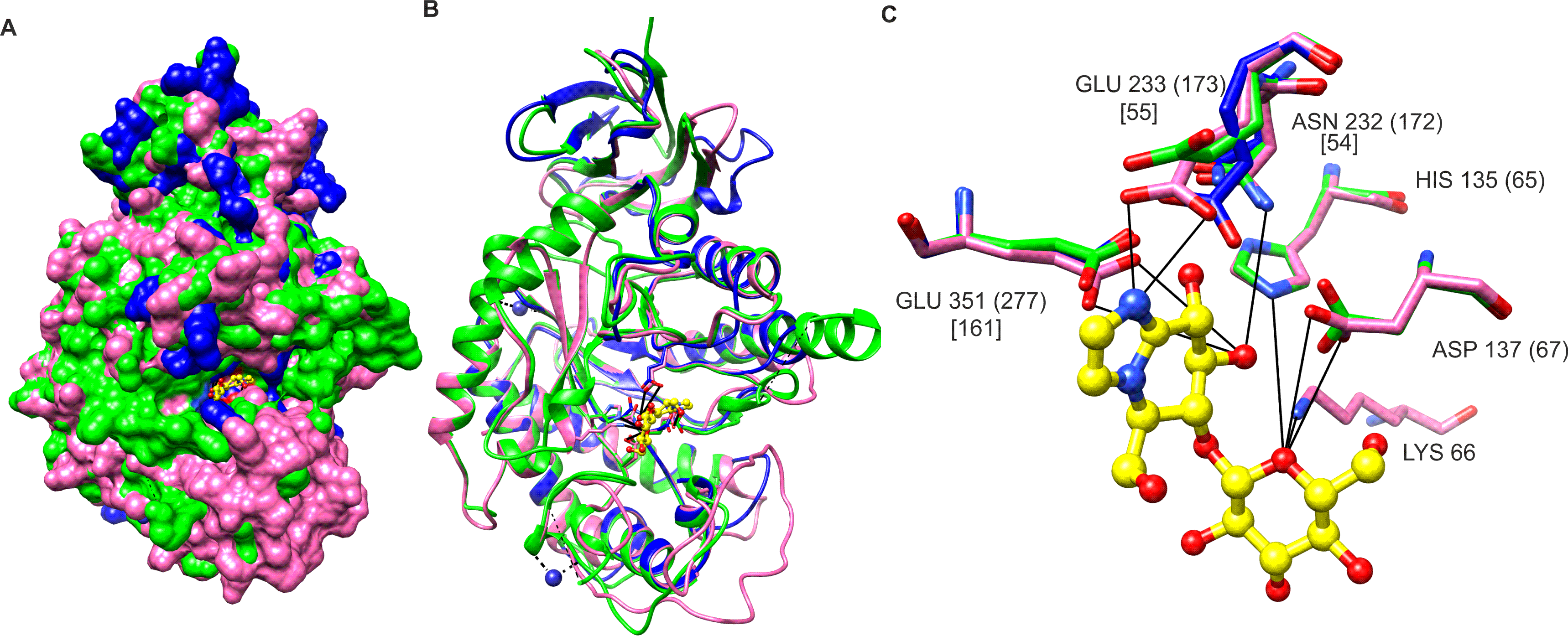
 **Supplementary figure 7.** Prediction of the shorter EGCase1 (DN1345_c0_g1_i1 DN11176_c0_g2_i1) structure and inhibitor binding site in *T. bryosalmonae*. The inhibitor (yellow) bound to the template is shown in ball and stick representation. The structures of parasite EGCases are superimposed (green and blue) on the chain B of the template endoglycoceramidase ii from *rhodococcus sp.* (pink). **A**) Similar binding cavities between template and targets can be seen around the inhibitor molecule. **B**) The same structure shown in ribbons to illustrate the consistency between template and target structures. **C)** The interactions between the EGCase II amino acids and inhibitor at the binding site are shown with black lines (Caines *et al.* 2007). The positions of the conserved amino acids at the binding site are in the template sequence (Caines *et al.* 2007) (after the amino acid), in the longer *T. bryosalmonae* EGCase2 (in parenthesis) and the shorter *T. bryosalmonae* EGCase1 (in square brackets).


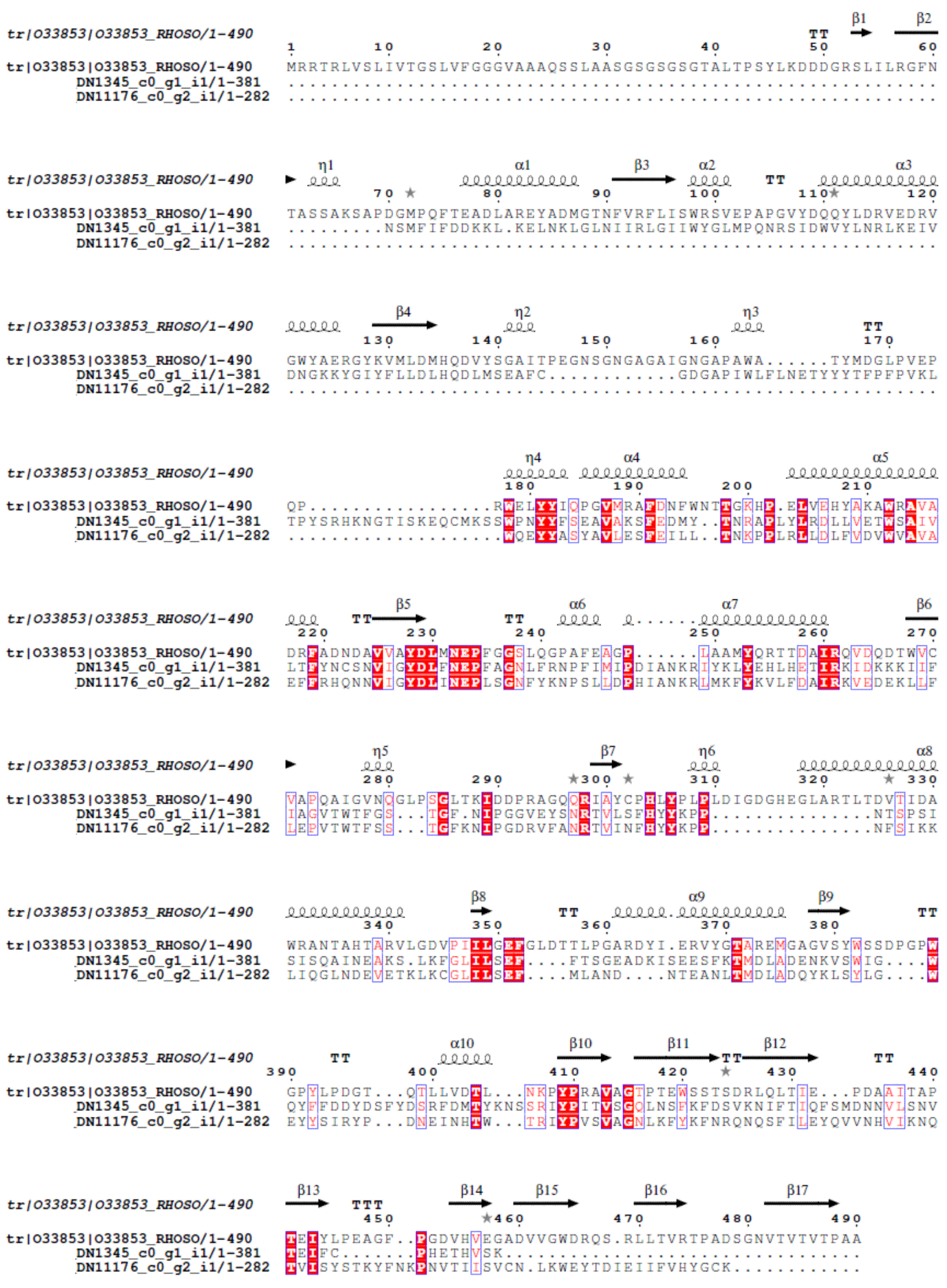


**Supplementary figure 8.** Multiple sequence alignment of the template (*rhodococcus sp.* endoglycoceramidase ii) and the two *T. bryosalmonae* EGCase sequences.


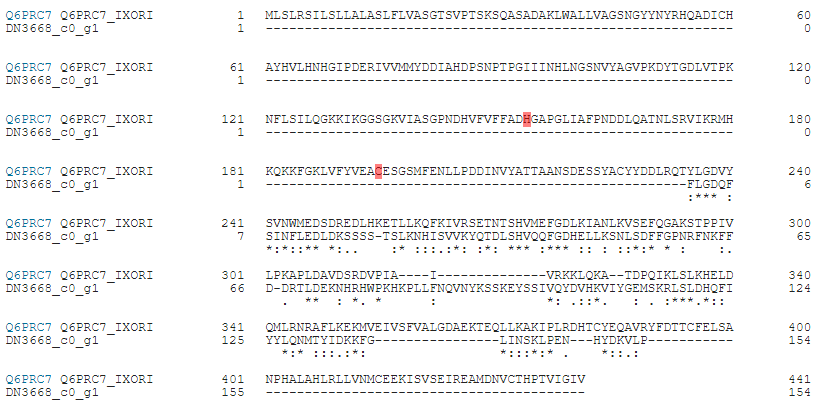


**Supplementary figure 9.** Sequence alignment of the ChEMBL Legumain-like protease (LGMN; CHEMBL1075261, UniProt ID: Q6PRC7) and the *T. bryosalmonae* partial legumain (DN3668_c0_g1). Alignment starts from 235th amino acid and does not cover the active site residues His(154) and Cys (195) (marked with red).


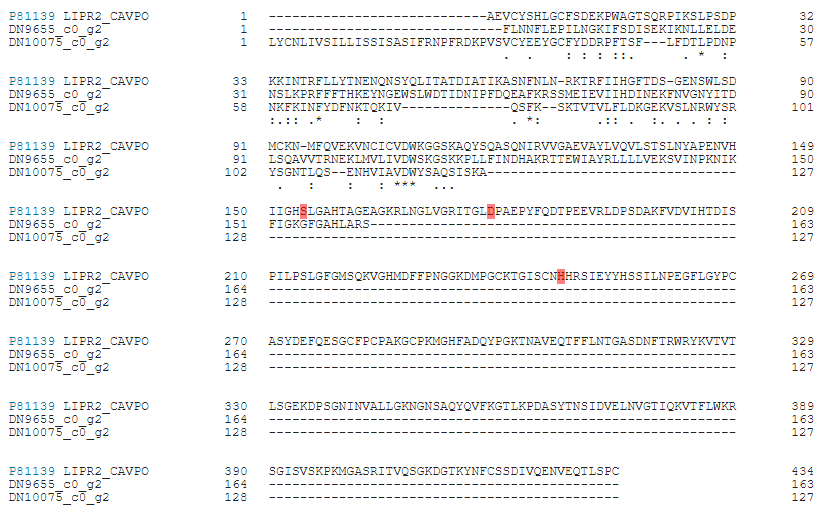


**Supplementary figure 10.** Sequence alignment of the ChEMBL Pancreatic lipase-related protein 2 (PLRP2; CHEMBL2169729, UniProt ID: P81139) and the *T. bryosalmonae* partial PLRP2s. The active site residues Ser(154) and A (178) and His (247) were not covered by the two *T. bryosalmonae* sequences.

**References:**

**Caines, MEC, Hancock, SM, Tarling, CA, Wrodnigg, TM, Stick, RV, Stütz, AE, Vasella, A, Withers, SG and Strynadka, NCJ** (2007) The Structural Basis of Glycosidase Inhibition by Five-Membered Iminocyclitols: The Clan A Glycoside Hydrolase Endoglycoceramidase as a Model System. *Angewandte Chemie International Edition* **46**, 4474–4476. doi: 10.1002/anie.200700268.

**Debes, PV, Gross, R and Vasemägi, A** 2017 Quantitative genetic variation in, and environmental effects on, pathogen resistance and temperature-dependent disease severity in a wild trout. *American Naturalist* **190**:244-265.
