# Supplementary figures and images for: Know your enemy – transcriptome of myxozoan Tetracapsuloides bryosalmonae reveals potential drug targets against proliferative kidney disease in salmonids
Source: Parasitology. 2021 Jan 22;148(6):726–39. doi: 10.1017/S003118202100010X (PMC8056827; doi:10.1017/S003118202100010X)

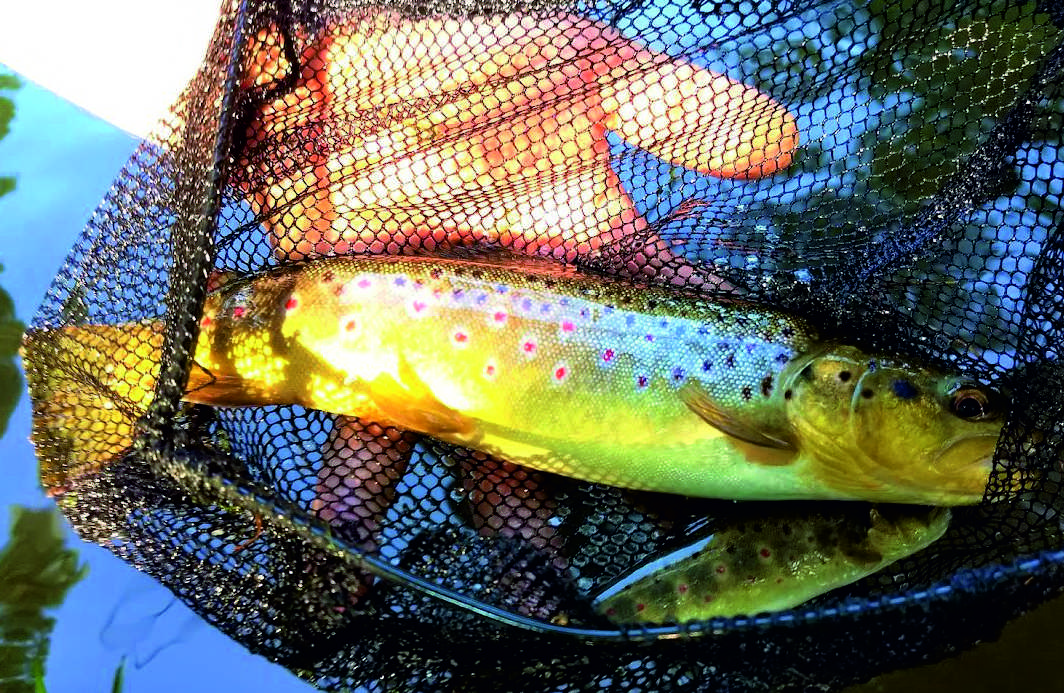

Supplement: Supplementary file 1 [file S003118202100010Xsup.zip › S003118202100010Xsup001.jpg]
